# The mechanochemical synthesis of quinazolin-4(3*H*)-ones by controlling the reactivity of IBX

**DOI:** 10.3762/bjoc.14.216

**Published:** 2018-09-12

**Authors:** Md Toufique Alam, Saikat Maiti, Prasenjit Mal

**Affiliations:** 1 School of Chemical Sciences, National Institute of Science Education and Research (NISER), HBNI, Bhubaneswar, PO Bhimpur-Padanpur, Via Jatni, District Khurda, Odisha 752050, India; Tel: +919439613856

**Keywords:** ball-mill, contact explosive, IBX, mechanochemical synthesis, quinazolin-4(3*H*)-one

## Abstract

Performing any synthesis using several arylamines and hypervalent iodine(V) reagents by direct mixing is unrealistic because of the high exothermic reaction or explosion. Herein we demonstrate, when anilines were substituted with an amide group at the *ortho*-position, successful chemical reactions could be performed due to intramolecular control. At maximum contact of the reacting substances, i.e.*,* under solvent-free mechanochemical conditions, 2-aminobenzamides, aryl-, alkylaldehydes and the iodine(V) reagent *o*-iodoxybenzoic acid (IBX) led to substituted quinazolin-4(3*H*)-one derivatives in fair yields.

## Introduction

An iodine and ammonia mixture is a well-known contact explosive due to formation of NI_3_ [1]. Similarly, hypervalent iodines as oxidizing compounds [2] react violently with amines under solvent-free conditions [3]. Aryliodonium imides or iminoiodanes can be prepared by the treatment of electron-deficient amines with iodine(III). However, these compounds explode at higher temperatures [4] and hence are stored under inert atmosphere and low temperature [5]. Polyvalent iodine derivatives are versatile reagents for C–N bond constructions [4,6]. Mechanochemical conditions such as ball milling are considered to be one of the premium techniques in solvent-free synthesis [7]. Under these conditions, maximum concentration is expected to put those systems under high stress and therefore violent exothermic reactions or even explosions may take place between hypervalent iodine reagents and electron-rich amines. For this reason, synthetic methods based on hypervalent iodine reagents and primary amines under solvent-free conditions or constrained media are limited [8]. Recently, we have described a method for the successful reaction of primary amines and hypervalent iodine(III) reagents by controlling the reactivity using an acid salt, NaHSO_4_, as additive [9].

## Results and Discussion

**Figure 1:**
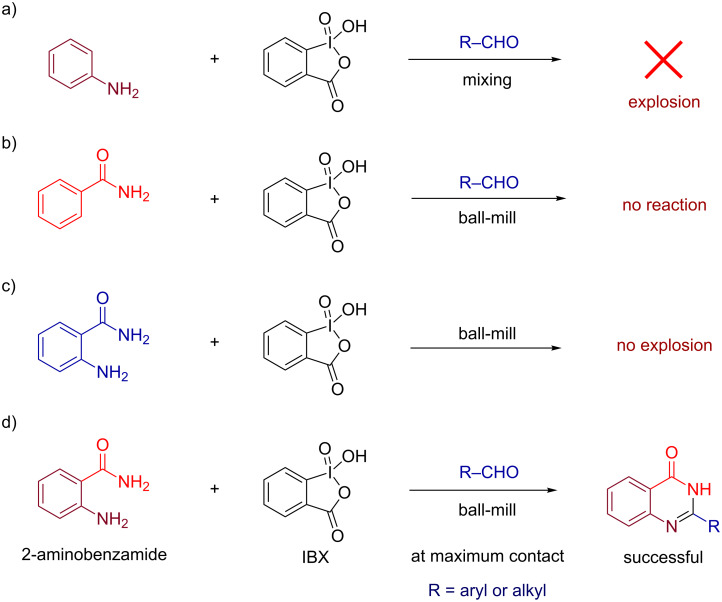
a) Explosion was observed when an arylamine was mixed with aldehydes in the presence of IBX. b) Benzamide was found to be unreactive with IBX. c) Treatment of 2-aminobenzamide with IBX did not lead to any explosion. d) Reaction of 2-aminobenzamide and aldehydes in the presence of IBX as oxidant afforded quinazolin-4(3*H*)-ones at maximum interaction of the reactants, i.e., under ball-milling conditions.

The last few decades have witnessed a significant growth in organic synthesis using hypervalent iodines [10–12]. Their easy availability, high stability, controlled oxidizing ability, and environmentally benign nature make them highly suitable for the development of new synthetic transformations [13–17]. The chemistry of iodine(V) reagents has been well documented in a number of reviews [18–20]. In continuation of our research focus on the development of synthetic methods using iodine-based reagents [21–26], we here report a method for the synthesis of quinazolin-4(3*H*)-ones [27–28] (Figure 1) from 2-aminobenzamide and aldehydes in the presence of *o*-iodoxybenzoic acid (IBX) [29]. However, when mixing benzaldehydes, aniline and IBX under ball-milling conditions an explosion was observed (Figure 1a; Caution! see experimental section) [30–31] and similar observations were made with Dess–Martin periodinane (DMP). On the other hand, benzamide was found to be unreactive with IBX and no reaction was observed under similar conditions (Figure 1b). Also no explosion was observed when 2-aminobenzamide was treated with IBX in absence of any aldehyde (Figure 1c). However, reacting 2-aminobenzamide with aldehydes in the presence of IBX under similar conditions was found to be successful affording the corresponding quinazolin-4(3*H*)-ones (Figure 1d).

**Figure 2:**
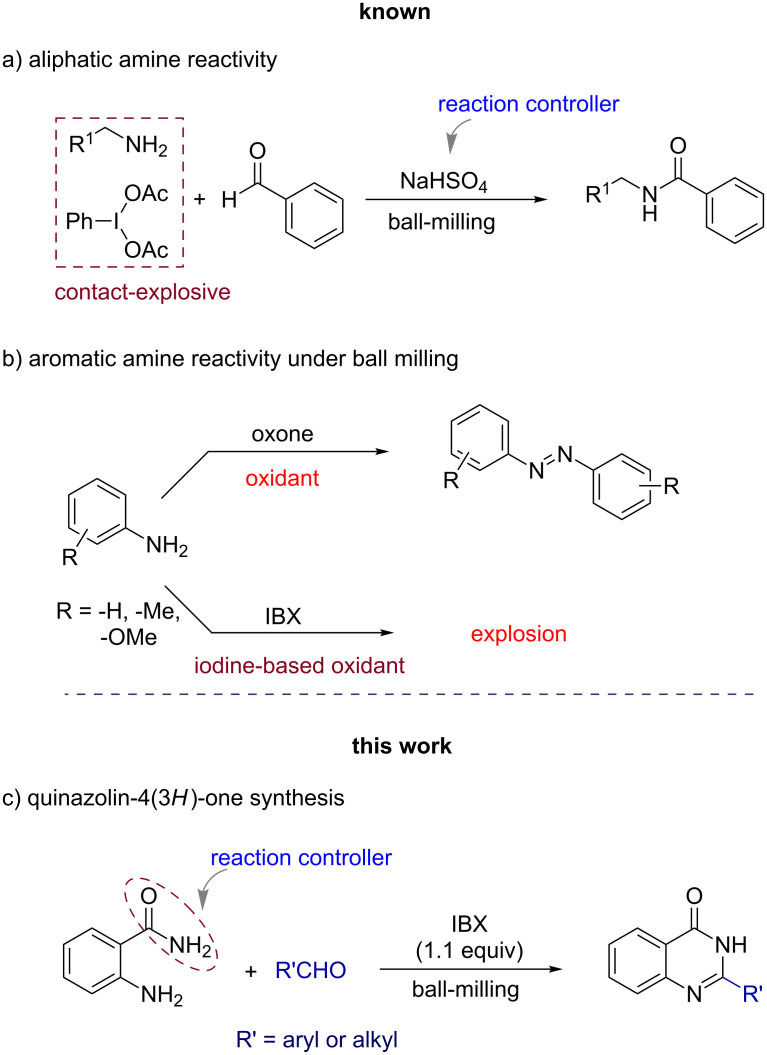
Comparison of the current work with the existing literature reports.

In a recent report we have shown a successful dehydrogenative cross-coupling or CDC reaction using a combination of primary amines and phenyleneiodine diacetate (PIDA) under solvent-free ball-milling conditions, i.e., at the highest possible contact of the reactants [9]. During the reaction with the stronger oxidant PIDA, the basicity of the amine was regulated using an externally added acid salt, NaHSO_4_ (Figure 2a). In Figure 2b, a comparison in the reactivities of arylamines in the presence of non-iodine-based oxidant oxone [32] and IBX (iodine-based oxidant) is shown. Anilines readily reacted with oxone leading to the formation of the azo derivatives [32], while their treatment with IBX led to explosive decomposition. The reactions of 2-aminobenzamide with arylaldehydes in the presence of IBX afforded quinazolin-4(3*H*)-ones at maximum contact of the reactants, i.e., under ball-milling conditions (Figure 2c).

Towards optimization of the reaction conditions, 2-aminobenzamide (**1**) and 4-ethylbenzaldehyde (**2a**) were chosen as model substrates (Table 1). Initially, 70% of 2-(4-ethylphenyl)quinazolin-4(3*H*)-one (**3a**) were obtained, when **1**, **2a** and 1.1 equiv of IBX were milled together for 1.5 h in a 10 mL ball-milling jar (Table 1, entry 1). However, the yield increased to 91% when IBX was added after 30 min of milling of the reactants **1** and **2a** (Table 1, entry 2). Conversely, changing the amount of IBX to other than 1.1 equiv, a decrease in the product yield was observed (Table 1, entries 3 and 4). The reaction also took place, albeit affording the product in lower yield, with in situ-generated IBX [33], i.e., by using an IBA (2-iodobenzoic acid)–oxone combination (Table 1, entry 5). Interestingly, when silica gel [34] was used as additive during the handling of liquid aldehydes, the yields of the products were found to be constant and reproducible. Interestingly no violent decomposition was observed, when IBX was added at the beginning of the reaction (Table 1, entry 1), after 30 min (Table 1, entry 2) or 1 h (Table 1, entry 6) of milling **1** and **2**. Thus we conclude that the current method is explosion free.

**Table 1:** Optimization of the reaction conditions.^a^

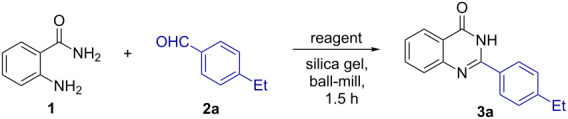		

Entry	Reagent (equiv)^b^	Yield (%)^c^

1^d^	IBX (1.1)	70
2	IBX (1.1)	91
3	IBX (1)	78
4	IBX (1.2)	85
5^e^	IBA (1.1)–oxone (1.5)	59
6^f^	IBX (1.1)	90

^a^Reaction conditions: **1a** (1 equiv) and **2a** (1 equiv) and 60 mg silica gel for approximately 60 µL of **2a**; ^b^reagent was added after 30 min; ^c^isolated yields. ^d^**1**, **2a** and IBX were added together; ^e^2-iodobenzoic acid (IBA). ^f^IBX added after 1 h.

**Figure 3:**
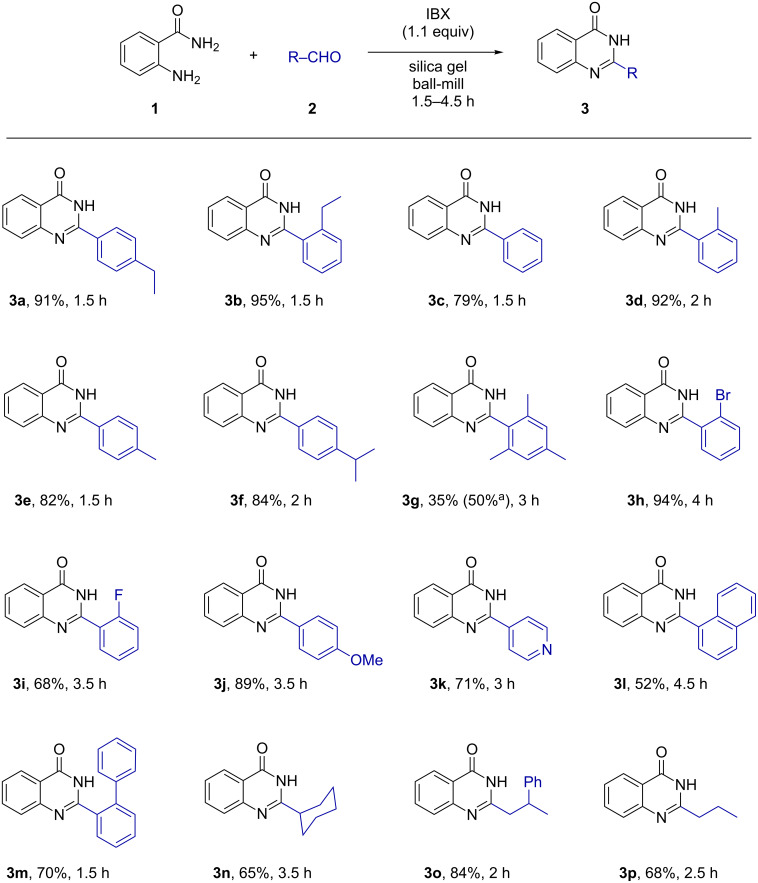
Synthesis of quinazolin-4(3*H*)-one derivatives from the reaction of **1** with liquid aldehydes. ^a^Yields with respect to recovered aldehydes, for compound **3k** IBX was added after 1 h.

**Figure 4:**
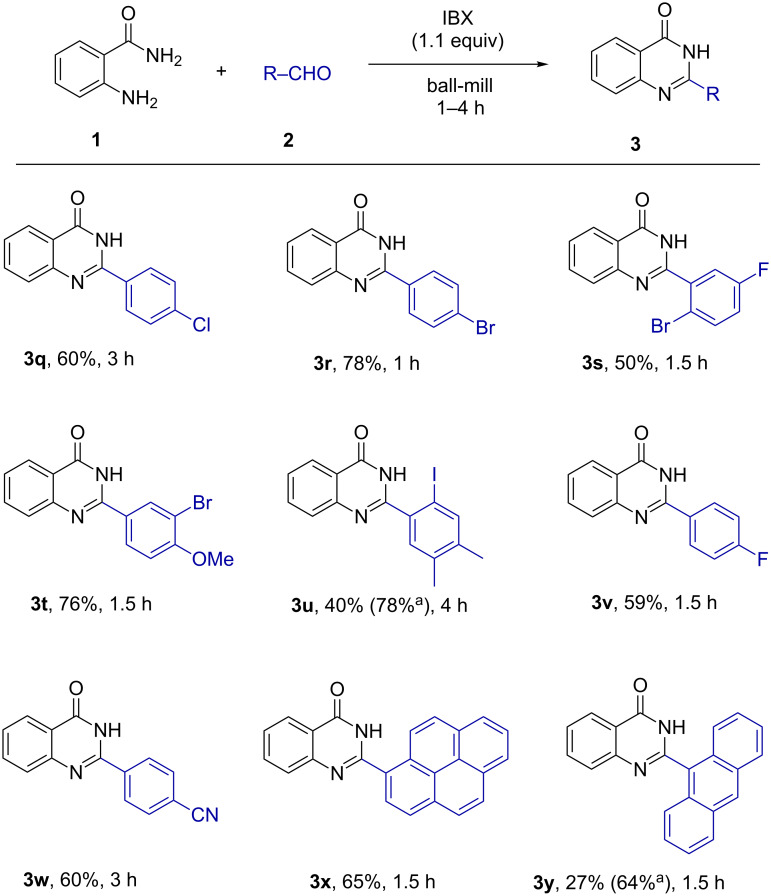
Synthesis of quinazolin-4(3*H*)-one derivatives from reaction of **1** and solid aldehydes. ^a^Yields with respect to recovered aldehydes, for compound **3y**, IBX was added after 1 h.

**Figure 5:**
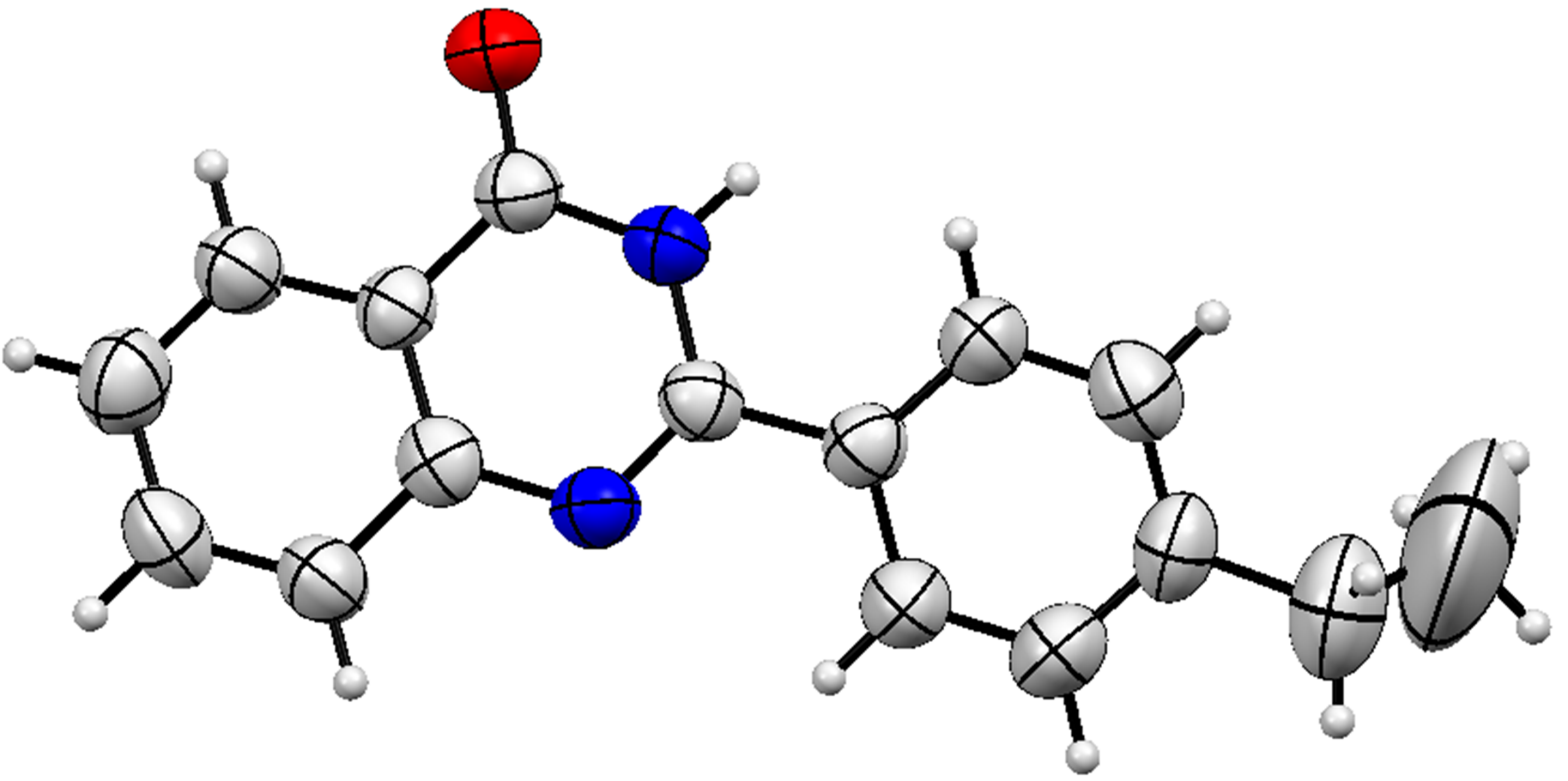
Crystal structure of **3a** (CCDC No. 1823611).

After optimization of the reaction conditions, the scope of the synthesis of quinazolin-4(3*H*)-one derivatives was explored (Figure 3 and Figure 4) and the desired products were isolated in fair yields. The yields of quinazolin-4(3*H*)-one derivatives were higher when monoalkyl-substituted benzaldehydes (**3a**,**b** and **3d**–**f**) were used compared to the reaction with unsubstituted benzaldehyde (**3c**). However, sterically congested aromatic aldehydes afforded the desired quinazolin-4(3*H*)-ones **3g** and **3u** in relatively poor yields. The reactions were found to proceed smoothly and moderately yielding with halogenated aldehydes (**3h**, **3i**, **3q–s**, **3v**) and the *p*-cyano-substituted aldehyde (**3w**). Methoxy-substituted aldehydes were giving quinazolin-4(3*H*)-ones in high yields (**3j**, **3t**). Likewise, the reaction was found to be successful with aldehydes containing fused aromatic ring systems like naphthyl (**3l**), pyrenyl (**3x**), anthryl (**3y**), etc. Several aliphatic aldehydes like cyclohexyl (**3n**), 3-phenylbutraldehyde (**3o**) and butyraldehyde (**3p**) furnished the corresponding heterocycles with higher efficiency. The synthesized products were characterized by standard analytical methods and the structure of **3a** was confirmed by X-ray crystallographic analysis (Figure 5).

**Figure 6:**
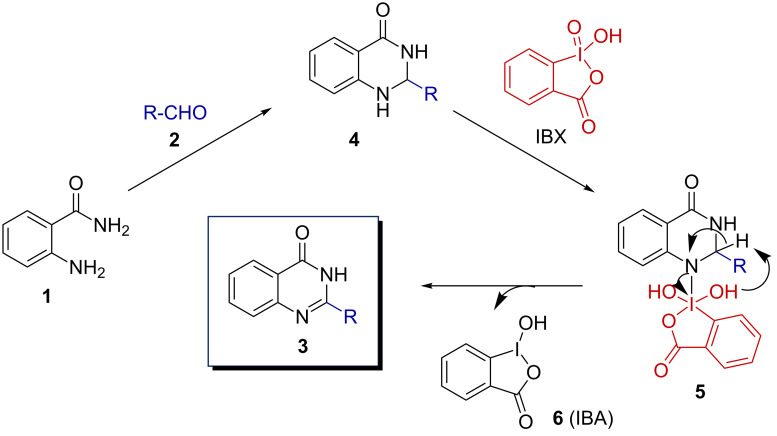
Plausible mechanism for the quinazolin-4(3*H*)-ones synthesis using IBX.

Based on the results collected in Table 1 and literature precedence [35–36], a mechanism of the quinazolin-4(3*H*)-ones synthesis is proposed and depicted in Figure 6. As it has been observed that the yield of the reaction significantly increased when IBX was added after 30 min of initial milling of the reactants **1** and **2** it is anticipated that 2-aminobenzamide and the arylaldehyde formed the adduct **4**. This intermediate then further reacted with IBX to generate **5**. Finally, **5** led to the quinazolin-4(3*H*)-one **3** with the generation of IBA (**6**).

Arylamines caused explosion and benzamide was found to be unreactive with IBX. However, the reaction was found to be successful with 2-aminobenzamide under similar conditions (Figure 1). 2-Aminobenzamide having one highly reactive amine part and another unreactive part, can be considered an aniline derivative with moderate reactivity. Therefore, we anticipate that the controlled reactivity of IBX under mechano-milling conditions led to successful reaction with 2-aminobenzamide.

**Scheme 1:**
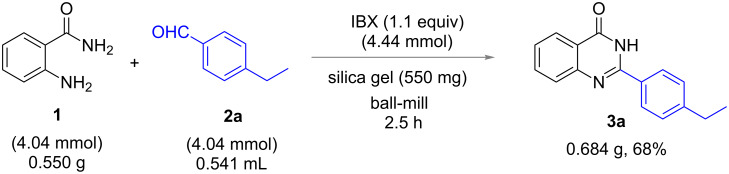
Large scale synthesis of **3a**.

Finally, a large scale synthesis was performed to prove the synthetic utility of this methodology. By taking anthranilamide (**1**, 0.550 g) and 4-ethylbenzaldehyde (**2a**, 0.541 mL), the reaction was carried out under optimized conditions. The 2-(4-ethylphenyl)quinazolin-4(3*H*)-one (**3a**) was isolated in 68% yield (Scheme 1).

## Conclusion

In conclusion, we have successfully demonstrated the synthesis of quinazolin-4(3*H*)-ones by the controlled use of IBX in the presence of 2-aminobenzamides. We foresee that controlled reactions of IBX in the presence of amines have certain advantages, e.g., many difficult reactions can be performed easily by choosing the appropriate reaction environment. The described methodology also highlights the advancement of quinazolin-4(3*H*)-ones synthesis chemistry and may find application in the context of mechanochemical reactions towards natural product synthesis and pharmaceutical chemistry.

## Experimental

**General methods.** Ball-milling experiments were carried out open to atmosphere and at room temperature in a Retsch MM 200 high speed vibration mixture milling instrument (21 Hz). All yields mentioned are isolated yields after column chromatographic purifications of the compounds using silica gel (230–400 mesh) and hexane/ethyl acetate mixtures as eluent, unless otherwise specified. NMR spectra were recorded on either a 400 MHz or a 700 MHz instrument at 25 °C. The chemical shift values are reported in ppm (parts per million) with respect to residual chloroform (7.26 ppm for ^1^H and 77.16 for ^13^C) or DMSO (2.5 ppm for ^1^H and 39.5 for ^13^C). Data are reported as follows: chemical shift in ppm (δ), multiplicity (s = singlet, d = doublet, t = triplet, q = quartet, brs = broad singlet, m = multiplet), coupling constant (Hz) and integration. High-resolution mass spectra (HRMS) were recorded on an ESI–TOF (time of flight) mass spectrometer. IR (infrared) spectral data are reported in wave numbers (cm^−1^). Melting points (mp) of the compounds were determined using a digital melting point apparatus and are uncorrected.

**Caution.** When aniline and IBX are mixed under solvent-free conditions or at maximum contact, immediate explosion was observed. However, no explosion could be observed under similar conditions when 2-aminobenzamide and arylaldehydes were reacted in the presence of IBX. However, it is highly recommended to consult the general safety protocols at the laboratory and all reactions should be carried out in a fume hood behind a blast shield.

2-Iodoxybenzoic acid (IBX) was prepared by following reported literature procedure [37].

**General procedure for preparation of quinazolin-4(3*H*)-ones.** 2-Aminobenzamide (**1**, 0.44 mmol, 1.0 equiv), aldehyde (**2**, 0.44 mmol, 1.0 equiv), 60 mg silica gel (only for liquid aldehydes) and a stainless-steel milling ball were added into a 10 mL stainless-steel jar. Milling was carried out for 30 min and then IBX (0.484 mmol, 1.1 equiv) was added to the mixture and milling was continued for 1 h. The progress of the reaction was monitored by TLC after taking a small portion of the reaction mixture and dissolving it in DCM (with the appropriate solvent as eluent). After completion of the reaction, dichloromethane was used for extracting the compound from the solid reaction mixture. The solvent was evaporated to dryness and the crude reaction mixture was purified by silica gel column chromatography using an appropriate hexane/ethyl acetate mixture.

**Large scale preparation of 3a:** One third of the 25 mL stainless steel milling jar was filled with 2-aminobenzamide (550 mg, 4.04 mmol), 4-ethylbenzaldehyde (541.5 µL, 4.04 mmol), 550 mg silica gel and one ball (15 mm diameter). After 1 h of milling, IBX (1.24 g, 4.44 mmol) was added and milling continued for 1.5 h. Then, after extraction of the reaction mixture with DCM, followed by silica gel column chromatography with ethyl acetate/hexane 1:5 as eluent provided product **3a** (684 mg, 68%).

## Supporting Information

File 1 Characterization data, NMR spectra and crystallographic information.
