# Supplementary material for: The mechanochemical synthesis of quinazolin-4(3H)-ones by controlling the reactivity of IBX
Source: Beilstein J Org Chem. 2018 Sep 12;14:2396–403. doi: 10.3762/bjoc.14.216 (PMC6142747; doi:10.3762/bjoc.14.216)
Supplement: File 1 — Characterization data, NMR spectra and crystallographic information. [file Beilstein_J_Org_Chem-14-2396-s001.pdf]

Supporting Information

for

The mechanochemical synthesis of quinazolin-4(3*H*)-ones by controlling the reactivity of IBX

Md Toufique Alam, Saikat Maiti and Prasenjit Mal*

Address: School of Chemical Sciences, National Institute of Science Education and Research (NISER), HBNI, Bhubaneswar, PO Bhimpur-Padanpur, Via Jatni, District Khurda, Odisha 752050, India; Tel: +919439613856

Email: Prasenjit Mal - pmal@niser.ac.in

*Corresponding author

Characterization data, NMR spectra and crystallographic information

Table of contents

Compound characterization data S2

Crystallographic data..S9

References...S11

NMR Spectra...S13

Compounds characterization data

2-(4-Ethylphenyl)quinazolin-4(3*H*)-one (3a) [1]. R_f = 0.5 (hexane/ethyl acetate 4:1); white solid; Yield: 91% (100 mg); ^{1}H NMR (700 MHz, $CDCl_3$) δ 11.38 (s, 1H), 8.33 (dd, J_1 = 7.7, J_2 =0.7 Hz, 1H), 8.16 (d, *J* = 8.4 Hz, 2H), 7.85 (d, *J* = 8.4 Hz, 1H), 7.82 – 7.78 (m, 1H), 7.50 (t, *J* = 7.7 Hz, 1H), 7.41 (d, *J* = 8.4 Hz, 2H), 2.76 (q, *J* = 7.7 Hz, 2H), 1.31 (t, *J* = 7.7 Hz, 3H); ^{13}C NMR (175 MHz, $CDCl_3$) δ 163.7, 152.1, 149.2, 148.9, 135.2, 129.8, 128.8, 127.8, 127.6, 126.9, 126.6, 120.8, 29.0, 15.4; HR-MS (ESI-TOF): m/z calculated for $C_{16}H_{14}NO_2$ $[M+H]^+$: 251.1179, found: 251.1200.

2-(2-Ethylphenyl)quinazolin-4(3*H*)-one (3b). R_f = 0.5 (hexane/ethyl acetate 7:3); white solid; Yield: 95% (105 mg); mp 145-147 ^{o} C; ^{1}H NMR (400 MHz, $CDCl_3$) δ 11.11 (s, 1H), 8.20 (d, *J* = 8 Hz, 1H), 7.79 (m, 2H), 7.55 (d, *J* = 7.6 Hz, 1H), 7.51 – 7.42 (m, 2H), 7.39 (d, *J* = 7.6 Hz, 1H), 7.34 (t, *J* = 7.6 Hz, 1H), 2.89 (q, *J* = 7.2 Hz, 2H), 1.22 (t, *J* = 7.6 Hz, 3H); ^{13}C NMR (100 MHz, $CDCl_3$) δ 163.4, 153.8, 149.3, 143.2, 134.5, 133.4, 130.8, 129.8, 128.9, 127.9, 127.1, 126.5, 126.3, 120.8, 26.9, 15.7; IR (KBr): ῡ = 1962, 2862, 2096, 1651, 1302, 1267, 1148 cm^{-1}; HR-MS (ESI-TOF): m/z calculated for $C_{16}H_{14}NO_2$ $[M+ H]^+$: 251.1179, found: 251.1191.

2-Phenylquinazolin-4(3*H*)-one (3c) [2]. R_f = 0.5 (hexane/ethyl acetate 4:1); white solid; Yield:79% (76mg); ^{1}H NMR (400 MHz, $CDCl_3$) δ 11.69 (s, 1H), 8.34 (d, *J* = 8 Hz, 1H), 8.27 (m, 2H), 7.86-7.79 (m, 2H), 7.60 (m, 3H), 7.51 (t, *J* = 7.2 Hz, 1H); ^{13}C NMR (100 MHz, $CDCl_3$) δ 163.9, 151.9, 149.7, 135.1, 132.9, 131.8, 129.2, 128.2, 127.5, 126.9, 126.5, 121.0

2-(*o*-Tolyl)quinazolin-4(3*H*)-one (3d) [2]. R_f = 0.4 (hexane: ethyl acetate 4:1); white solid; Yield: 92% (96 mg) ; ^{1}H NMR (400 MHz, $CDCl_3$) δ 10.79 (s, 1H), 8.24 (d, *J* = 8 Hz, 1H),

7.80 (d, J = 4 Hz, 2H), 7.57 (d, J = 7.2 Hz, 1H), 7.50 (dt, J_1 = 8, J_2 = 4 Hz, 1H), 7.43 (t, J = 7.2 Hz, 1H), 7.35 (d, J = 7.2 Hz, 2H), 2.53 (s, 3H); ^{13}C NMR (100 MHz, $CDCl_3$) δ 163.14, 153.6, 149.2, 137.0, 134.9, 133.7, 131.6, 130.7, 128.9, 127.9, 127.1, 126.5, 126.4, 120.9, 20.2.

2-(*p*-Tolyl)quinazolin-4(3*H*)-one (3e) [2]. R_f = 0.5 (hexane/ethyl acetate 4:1); white solid; Yield: 82% (86 mg); ^{1}H NMR (700 MHz, DMSO-d_6) δ 12.46 (s, 1H), 8.14 (d, J = 7.7 Hz, 1H), 8.10 (d, J = 8.4 Hz, 2H), 7.83 (t, J = 7.7 Hz, 1H), 7.73 (d, J = 7.7 Hz, 1H), 7.51 (t, J = 7.7 Hz, 1H), 7.36 (d, J = 7.7 Hz, 2H), 2.39 (s, 3H); ^{13}C NMR (175 MHz, DMSO-d_6) δ 162.3, 152.3, 148.8, 141.5, 134.6, 129.9, 129.2, 127.7, 127.4, 126.4, 125.9, 21.0; HR-MS (ESI-TOF): m/z calculated for $C_{15}H_{12}NO_2$ $[M+ H]^+$: 237.1022, found: 237.1026.

2-(4-Isopropylphenyl)quinazolin-4(3*H*)-one (3f) [2]. R_f = 0.5 (hexane/ethyl acetate 4:1); white solid; Yield: 84% (97 mg); ^{1}H NMR (400 MHz, $CDCl_3$) δ 11.73 (s, 1H), 8.34 (d, J = 7.6 Hz, 1H), 8.21 (d, J = 8 Hz, 2H), 7.89 – 7.74 (m, 2H), 7.57 – 7.47 (m, 1H), 7.44 (d, J = 8.4 Hz, 2H), 3.15 – 2.91 (m, 1H), 1.32 (d, J = 6.8 Hz, 6H); ^{13}C NMR (100 MHz, $CDCl_3$) δ 164.1, 153.2, 151.9, 149.8, 134.9, 130.4, 128.1, 127.6, 127.3, 126.7, 126.5, 120.9, 34.3, 23.9.

2-Mesitylquinazolin-4(3*H*)-one (3g) [3]. R_f = 0.5 (hexane/ethyl acetate 4:1); white solid; Yield: 35% (40 mg), 50% (based on recovered aldehyde); ^{1}H NMR (400 MHz, DMSO-d_6) δ 12.42 (s, 1H), 8.17 (d, J = 7.6 Hz, 1H), 7.83 (t, J = 7.6 Hz, 1H), 7.67 (d, J = 8.0 Hz, 1H), 7.54 (t, J = 7.6 Hz, 1H), 6.97 (s, 2H), 2.29 (s, 3H), 2.12 (s, 6H); ^{13}C NMR (100 MHz, DMSO-d_6) δ 161.8, 154.3, 148.9, 138.4, 135.4, 134.5, 132.0, 128.0, 127.4, 126.7, 125.9, 121.1, 20.8, 19.1.

2-(2-Bromophenyl)quinazolin-4(3*H*)-one (3h) [4]. R_f = 0.4 (hexane/ethyl acetate 4:1); white solid; Yield: 94% (126 mg); ^{1}H NMR (400 MHz, $CDCl_3$) δ 10.60 (s, 1H), 8.26 (d, *J* = 8 Hz, 1H), 7.82 (d, *J* = 4 Hz, 2H), 7.71 (t, *J* = 7.2 Hz, 2H), 7.57 – 7.49 (m, 1H), 7.47 (d, *J* = 7.2 Hz, 1H), 7.40 (t, *J* = 8 Hz, 1H); ^{13}C NMR (100 MHz, $CDCl_3$) δ 162.6, 152.2, 149.1, 135.3, 135.0, 133.9, 132.1, 131.4, 128.1, 128.1, 127.5, 126.6, 121.2, 121.1.

2-(2-Fluorophenyl)quinazolin-4(3*H*)-one (3i) [5]. R_f = 0.4 (hexane/ethyl acetate 4:1); white solid; Yield: 68% (72 mg); ^{1}H NMR (400 MHz, $CDCl_3$) δ 10.37 (s, 1H), 8.30 (m, 2H), 7.81 (m, 2H), 7.52 (m, 1H), 7.52 (d, *J* = 8 Hz, 1H), 7.35 (t, *J* = 8 Hz, 1H),7.23 (t, *J* = 8 Hz,1H); ^{13}C NMR (100 MHz, $CDCl_3$) δ 162.2, 160.9 (d, $^1J_{C, F}$ = 249.9 Hz), 149.1, 148.6 (d, $^4J_{C, F}$ = 1.6 Hz), 134.9, 133.6 (d, $^3J_{C, F}$ = 9.2 Hz), 131.5 (d, $^4J_{C, F}$ = 2.0 Hz), 128.13, 127.3, 126.7, 125.3 (d, $^4J_{C, F}$ = 3.3 Hz), 121.3, 120.4 (d, $^3J_{C, F}$ = 9.3 Hz), 116.8 (d, $^2J_{C, F}$ = 23 Hz).

2-(4-Methoxyphenyl)quinazolin-4(3*H*)-one (3j) [2]. R_f = 0.5 (hexane/ethyl acetate 4:1); white solid; Yield: 89% (99 mg); ^{1}H NMR (400 MHz, DMSO-d_6) δ 12.41 (s, 1H), 8.19 (d, *J* = 7.6 Hz, 2H), 8.13 (d, *J* = 7.6 Hz, 1H), 7.79 (t, *J* = 7.2 Hz, 1H), 7.69 (d, *J* = 8.0 Hz, 1H), 7.47 (t, *J* = 7.2 Hz, 1H), 7.08 (d, *J* = 7.6 Hz, 2H), 3.84 (s, 3H); ^{13}C NMR (100 MHz, DMSO-d_6) δ 162.4, 162.1, 151.9, 148.9, 134.5, 129.5, 127.3, 126.1, 125.9, 124.8, 120.7, 114.0, 55.5.

2-(Pyridin-4-yl)quinazolin-4(3*H*)-one (3k) [6]. R_f = 0.3 (hexane/ethyl acetate 1:1); white solid; Yield: 71 % (70 mg); ^{1}H NMR (400 MHz, DMSO-d_6) δ 12.79 (s, 1H), 8.79 (d, *J* = 5.2 Hz, 1H), 8.18 (d, *J* = 7.6 Hz, 1H), 8.12 (d, *J* = 6 Hz, 2H), 7.88 (t, *J* = 8 Hz, 1H), 7.80 (d, *J* = 8.0 Hz, 1H), 7.59 (t, *J* = 7.6 Hz, 1H); ^{13}C NMR (100 MHz, DMSO-d_6) δ 162.2, 150.7, 150.4, 148.4, 139.9, 134.9, 127.8, 127.5, 125.9, 121.7. 121.5.

2-(Naphthalen-1-yl)quinazolin-4(3*H*)-one (3l) [7]. R_f = 0.4 (hexane/ethyl acetate 7:3); white solid; Yield: 52% (62 mg); ^{1}H NMR (400 MHz, DMSO-d$_6$) δ 12.66 (s, 1H), 8.23 (d, *J* = 8 Hz, 1H), 8.47 (d, *J* = 8.8, 1H), 8.13 (d, *J* = 8 Hz, 1H), 8.07 – 8.03 (m, 1H), 7.90 – 7.84 (m, 1H), 7.80 (d, *J* = 6.8 Hz, 1H), 7.74 (d, *J* = 8 Hz, 1H), 7.68 – 7.63 (m, 1H), 7.62 – 7.56 (m, 3H); ^{13}C NMR (100 MHz, DMSO-d$_6$) δ 161.9, 153.7, 148.7, 134.5, 133.1, 131.7, 130.3, 130.3, 128.3, 127.7, 127.5, 127.0, 126.8, 126.3, 125.8, 125.2, 125.0, 121.2; HR-MS (ESI-TOF): m/z calculated for $C_{18}H_{12}NO_2$ [M+ H] $^+$: 273.1022, found: 273.1039.

2-([1,1'-biphenyl]-2-yl)quinazolin-4(3*H*)-one (3m) [8]. R_f = 0.4 (hexane/ethyl acetate 4:1); white solid; Yield: 70% (92 mg); ^{1}H NMR (400 MHz, $CDCl_3$) δ 9.43 (s, 1H), 8.17 (d, *J* = 7.6 Hz, 1H), 7.87 (d, *J* =7.6, 1H), 7.78 (m, 2H), 7.58 (t, *J* = 7.6 Hz, 1H), 7.49 (d, *J* =7.6 Hz, 3H), 7.32-7.26 (m, 5H); ^{13}C NMR (100 MHz, $CDCl_3$) δ 161.9, 153.7, 149.2, 140.7, 139.3, 134.8, 132.8, 131.1, 131.0, 130.5, 129.1, 128.9, 128.2, 128.1, 127.9, 127.1, 126.5, 120.8.

2-Cyclohexylquinazolin-4(3*H*)-one (3n) [9]. R_f = 0.5 (hexane/ethyl acetate 3:2); white solid; Yield: 65% (65 mg); ^{1}H NMR (400 MHz, $CDCl_3$) δ 10.86 (s, 1H), 8.27 (d, *J* = 7.6 Hz, 1H), 7.76 (t, *J* = 8, 1H), 7.70 (d, *J* = 8, 1H) 7.46 (t, *J* = 7.6 Hz, 1H), 2.70 (t, *J* = 12 Hz, 1H), 2.06 (d, *J* = 11.6 Hz, 2H), 1.92 (d, *J* = 12.8 Hz, 2H), 1.87 – 1.65 (m, 3H), 1.54 – 1.35 (m, 3H); ^{13}C NMR (100 MHz, $CDCl_3$) δ 164.5, 160.4, 149.7, 134.9, 127.5, 126.4, 126.3, 120.9, 44.9, 30.6, 26.1, 25.8.

2-(2-Phenylpropyl)quinazolin-4(3*H*)-one (3o). R_f = 0.5 (hexane/ethyl acetate 7:3); white solid; Yield: 84% (102 mg); mp 165-166 ^{o}C; ^{1}H NMR (400 MHz, $CDCl_3$) δ 12.05 (s, 1H), 8.33 (d, *J* = 7.6 Hz, 1H), 7.79 (m, 2H), 7.52 (t, *J* = 7.2 Hz, 1H), 7.37 (d, *J* = 6.8 Hz, 2H), 7.28 (d, *J* = 6.4 Hz, 2H), 7.20 (d, *J* = 6.8 Hz, 1H), 3.55 (m, 1H), 3.19 – 2.94 (m, 2H), 1.42 (d, *J* =

6.4 Hz, 3H); ^{13}C NMR (100 MHz, $CDCl_3$) δ 164.4, 155.7, 149.6, 145.5, 134.9, 128.7, 127.5, 127.1, 126.7, 126.6, 126.4, 120.6, 44.7, 38.8, 21.1; IR (KBr): ῡ = 2973, 2923, 2103, 1653, 1467, 1335, 1251 cm^{-1}; HR-MS (ESI-TOF): m/z calculated for $C_{17}H_{16}NO_2$ $[M+ H]^+$: 265.1335, found: 265.1361.

2-Propylquinazolin-4(3*H*)-one (3p) [10]. R_f = 0.3 (hexane/ethyl acetate 7:3); white solid; Yield: 68% (57 mg); ^1H NMR (400 MHz, DMSO-d_6) δ 12.18 (s, 1H), 8.07 (d, *J* = 7.6 Hz, 1H), 7.77 (t, *J* = 6.8 Hz, 1H), 7.59 (d, *J* = 7.6 Hz, 1H), 7.45 (t, *J* = 7.1 Hz, 1H), 2.57 (t, *J* = 6.4 Hz, 2H), 1.74 (m, 2H), 0.93 (t, *J* = 6.8 Hz, 3H); ^{13}C NMR (100 MHz, DMSO-d_6) δ 161.9, 157.3, 148.9, 134.3, 126.8, 125.9, 125.7, 120.8, 36.4, 20.2, 13.5.

2-(4-Chlorophenyl)quinazolin-4(3*H*)-one (3q) [4]. R_f = 0.5 (hexane/ethyl acetate 4:1); white solid; Yield: 60% (67 mg); ^1H NMR (400 MHz, DMSO-d_6) δ 12.62 (s, 1H), 8.20 (d, *J* = 8.4 Hz, 2H), 8.14 (d, *J* = 8, 1H), 7.85 (t, *J* = 7.6 Hz, 1H), 7.74 (d, *J* = 8.0 Hz, 1H), 7.63 (d, *J* = 8.0 Hz, 2H), 7.53 (t, *J* = 7.6 Hz, 1H); ^{13}C NMR (100 MHz, DMSO-d_6) δ 161.7, 151.4, 148.6, 136.3, 134.7, 131.6, 129.6, 128.7, 127.5, 126.8, 125.9, 120.9; HR-MS (ESI-TOF): m/z calculated for $C_{14}H_9NO_2Cl$ $[M+ H]^+$: 257.0476, found: 257.0479.

2-(4-Bromophenyl)quinazolin-4(3*H*)-one (3r) [4]. R_f = 0.6 (hexane/ethyl acetate 4:1); white solid; Yield: 78% (104 mg); ^1H NMR (400 MHz, DMSO-d_6) δ 12.60 (s, 1H), 8.13 (d, *J* = 8.4 Hz, 3H), 7.84 (t, *J* = 6.4, 1H), 7.76 (d, *J* = 6.4 Hz, 3H), 7.53 (t, *J* = 6.4, 1H); ^{13}C NMR (175 MHz, DMSO-d_6) δ 162.2, 151.5, 148.6, 134.7, 131.9, 131.7, 129.8, 127.5, 126.8, 125.9, 125.2, 121.0.

2-(2-Bromo-5-fluorophenyl)quinazolin-4(3*H*)-one (3s). R_f = 0.5 (hexane/ethyl acetate 7:3); white solid; Yield: 50% (69 mg); mp 229-230 °C; ^{1}H NMR (400 MHz, $CDCl_3$) δ 10.56 (s, 1H), 8.28 (d, J = 8 Hz, 1H), 7.82 (m, 2H), 7.68 (dd, J_1 = 8.8, J_2 = 4 Hz, 1H), 7.61 – 7.49 (m, 2H), 7.16 (dd, J_1 = 8.4, J_2 = 5.6 Hz, 1H); ^{13}C NMR (100 MHz, $CDCl_3$) δ 162.4, 162.0 (d, $^1J_{C,F}$ = 250.3), 150.9, 148.9, 136.5 (d, $^3J_{C,F}$ = 7.9 Hz), 135.5 (d, $^3J_{C,F}$ = 7.9 Hz), 135.2, 128.2, 127.8, 126.7, 121.3, 119.6 (d, $^2J_{C,F}$ = 22.3 Hz), 118.9 (d, $^2J_{C,F}$ =24.6 Hz), 115.3 (d, $^4J_{C,F}$ = 3.5 Hz); IR (KBr): ʋ = 3427, 2096, 1651, 1338, 1251, 1203 cm^{-1}; HR-MS (ESI-TOF): m/z calculated for $C_{14}H_8NO_2{}^{79}BrF$ [M + H]$^+$: 318.9877, found: 318.9904, $C_{14}H_8NO_2{}^{81}Br$ [M + H] $^+$: 320.9857, found: 320.9885.

2-(3-Bromo-4-methoxyphenyl)quinazolin-4(3*H*)-one (3t). R_f = 0.5 (hexane/ethyl acetate 7:3); white solid; Yield: 76% (110 mg); mp charred at 250 °C; ^{1}H NMR (400 MHz, DMSO-d_6) δ 12.50 (s, 1H), 8.47 (s, 1H), 8.25 (d, J = 8 Hz, 1H), 8.13 (d, J = 8 Hz, 1H), 7.83 (t, J = 8.4 Hz, 1H), 7.73 (d, J = 8 Hz, 1H), 7.51 (t, J = 7.6 Hz, 1H), 7.28 (d, J = 8.4 Hz, 1H); ^{13}C NMR (100 MHz, DMSO-d_6) δ 162.3, 158.0, 150.8, 148.7, 134.7, 132.4, 128.8, 127.5, 126.5, 126.2, 125.9, 120.7, 112.7, 110.73, 56.7; IR (KBr): ʋ = 3193, 2089, 1643, 1224, 1141, 1055 cm^{-1}; HR-MS (ESI-TOF): m/z calculated for $C_{15}H_{11}NO_2{}^{79}Br$ [M + H] $^+$: 331.0077, found: 331.0081, $C_{15}H_{11}NO_2{}^{81}Br$ [M + H] $^+$: 333.0057, found: 333.0059.

2-(2-Iodo-4,5-dimethylphenyl)quinazolin-4(3*H*)-one (3u). R_f = 0.4 (hexane/ethyl acetate 4:1); white solid; Yield: 40% (66 mg), 78% (based on recovered aldehyde) ; mp 215-216 °C; ^{1}H NMR (400 MHz, DMSO-d_6) δ 12.49 (s, 1H), 8.17 (d, J = 7.6 Hz, 1H), 7.85 (t, J = 7.2 Hz, 1H), 7.76 (s, 1H), 7.69 (d, J = 8 Hz, 1H), 7.56 (t, J = 7.2 Hz, 1H), 7.35 (s, 1H), 2.26 (s, 3H), 2.23 (s, 3H); ^{13}C NMR (100 MHz, DMSO-d_6) δ 161.6, 155.2, 148.6, 140.5, 139.3, 137.1, 136.6, 134.6, 130.8, 127.5, 126.9, 125.9, 121.2, 92.5, 18.8, 18.7; IR (KBr): ʋ = 2873, 2090,

1644, 1463, 1288, 1138, 1020 cm^{-1}; HR-MS (ESI-TOF): m/z calculated for $C_{16}H_{13}NO_2I$ [M+H]$^+$: 377.0145, found: 377.0174.

2-(4-Fluorophenyl)quinazolin-4(3*H*)-one (3v) [2]. R_f = 0.5 (hexane/ethyl acetate 7:3); white solid; Yield: 59% (62 mg); ^{1}H NMR (400 MHz, DMSO-d$_6$) δ 12.59 (s, 1H), 8.25 (t, *J* = 8.0, Hz, 2H), 8.15 (d, *J* = 7.6 Hz, 1H), 7.84 (t, *J* = 7.6 Hz, 1H), 7.74 (d, *J* = 8.0 Hz, 1H), 7.52 (t, *J* = 7.2 Hz, 1H), 7.40 (t, *J* = 8.4 Hz, 2H); ^{13}C NMR (100 MHz, DMSO-d$_6$) δ 164.0 (d, $^1J_{C, F}$ = 247.0 Hz), 161.9, 150.7, 148.3, 134.7, 130.4 (d, $^3J_{C, F}$ = 9.1 Hz), 129.3 (d, $^4J_{C, F}$ = 2.5 Hz), 127.5, 126.7, 125.9, 120.7, 115.7 (d, $^2J_{C, F}$ =22.0 Hz).

4-(4-Oxo-3,4-dihydroquinazolin-2-yl)benzonitrile (3w) [7]. R_f = 0.4 (hexane/ethyl acetate 4:1); white solid; Yield: 60% (65 mg); ^{1}H NMR (400 MHz, DMSO-d$_6$) δ 12.76 (s, 1H), 8.34 (d, *J* = 8.4 Hz, 2H), 8.18 (d, *J* = 8 Hz, 1H), 8.04 (d, *J* = 8 Hz, 2H), 7.87 (t, *J* = 7.2 Hz, 1H), 7.78 (d, *J* = 8.0 Hz, 1H), 7.57 (t, *J* = 7.2 Hz, 1H); ^{13}C NMR (100 MHz, DMSO-d$_6$) δ 162.2, 151.1, 148.2,137.0, 134.8, 132.6, 128.7, 127.7, 127.2,125.9, 121.3, 118.4, 113.6.

2-(Pyren-1-yl)quinazolin-4(3*H*)-one (3x): R_f = 0.4 (hexane/ethyl acetate 7:3); yellow solid; Yield: 65% (99 mg); mp charred at 250 ^{o}C ; ^{1}H NMR (400 MHz, DMSO-d$_6$) δ 12.84 (s, 1H), 8.48 (d, *J* = 9.2 Hz, 1H), 8.43 (d, *J* = 8.0 Hz, 1H), 8.42 – 8.36 (m, 2H), 8.34 – 8.32 (m, 2H), 8.30 – 8.25 (m, 3H), 8.15 (t, *J* = 7.6 Hz, 1H), 7.90 (t, *J* = 8.4, 1H), 7.81 (d, *J* = 8.0 Hz, 1H), 7.62 (t, *J* = 7.6 Hz, 1H); ^{13}C NMR (100 MHz, DMSO-d$_6$) δ 162.1, 154.1, 148.9, 134.6, 131.9, 130.8, 130.2, 128.9, 128.8, 128.6, 128.5, 127.6, 127.3 (2C), 126.9, 126.8, 126.1, 125.9, 125.8, 124.5 (2C), 123.8, 123.6, 121.3; IR (KBr): $\bar{\upsilon}$ = 3308, 2862, 2089, 1636, 1278, 1148 cm^{-1}; HR-MS (ESI-TOF): m/z calculated for $C_{24}H_{14}NO_2$ [M+ H] $^+$: 347.1179, found: 347.1199.

2-(Anthracen-9-yl)quinazolin-4(3*H*)-one (3y) [11]. R_f = 0.4 (hexane/ethyl acetate 7:3); white solid; Yield: 27% (34 mg), 64% (based on recovered aldehyde); ^{1}H NMR (400 MHz, $CDCl_3$) δ 9.27 (s, 1H), 8.63 (s, 1H), 8.41 (d, *J* = 7.6 Hz, 1H), 8.12 – 8.04 (m, 2H), 7.92 – 7.84 (m, 4H), 7.63 (m, Hz, 1H), 7.51 (m, 4H); ^{13}C NMR (100 MHz, $CDCl_3$) δ 162.1, 152.2, 149.1, 135.2, 131.2, 130.1, 129.7, 128.9, 128.3, 127.7, 127.6, 127.2, 126.8, 125.8, 124.6, 121.4.

Crystallographic data collection

Good quality crystals of the compounds were obtained after slow evaporation of chloroform and hexane (1:1) mixture. The crystal data were collected with a Bruker SMART D8 goniometer equipped with an APEX CCD detector and with an INCOATEC micro source (MoKα radiation, λ = 0.71073 Å). SAINT+ [12] and SADABS [13] were used to integrate the intensities and to correct the absorption respectively. The structure was resolved by direct methods and refined on F^2 with SHELXL-97 [14].

Crystallographic Data

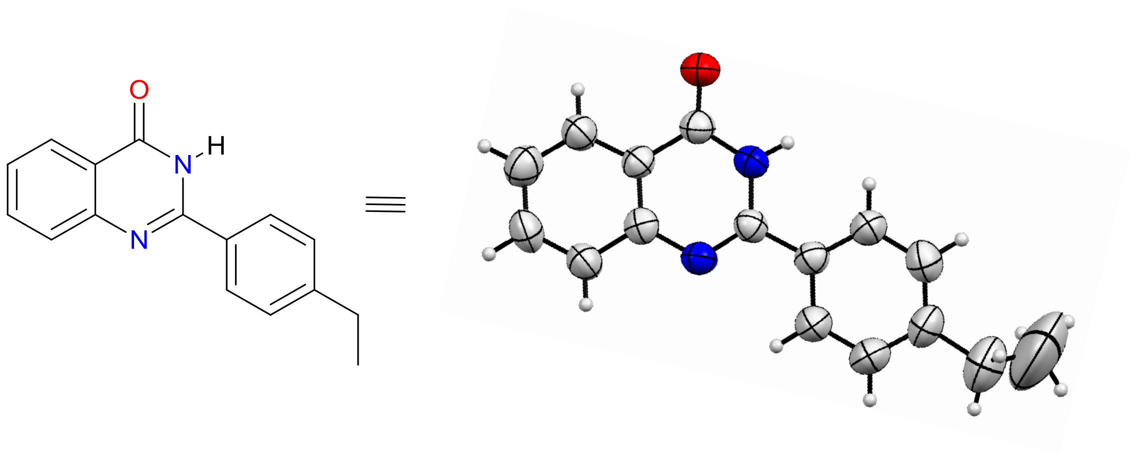

Figure S1: Crystal structure of **3a**

Table S1: Crystal data and structure refinement for 3a.

**CCDC No.**	**1823611**
Empirical formula	$C_{32}H_{28}N_4O_2$
Formula weight	500.58
Temperature	296.15 K
Crystal system	triclinic
Space group	P-1
Unit cell dimensions	a = 5.0503(3) Å α = 92.215(6)°
	b = 15.3328(10) β = 91.359(4)°
	c = 17.0240(10) γ = 99.349(4)°
Volume	1299.17(14) $Å^3$
Z	2
Density (calculated)	1.280 g/cm^3
Absorption coefficient	0.081 mm^{-1}
F(000)	528.0
Crystal size	0.31 × 0.26 × 0.21 mm^3
Radiation	MoKα (λ = 0.71073)
Theta range for data collection	2.396 to 50.922°
Index ranges	-6 ≤ h ≤ 6, -18 ≤ k ≤ 18, -20 ≤ l ≤ 20
Reflections collected	14947
Independent reflections	4773 [R_{int} = 0.0653, R_{sigma} = 0.1250]
Data/restraints/parameters	4773/3/345
Goodness-of-fit on F^2	0.971
Final R indexes [I>=2σ (I)]	R_1 = 0.0857, wR_2 = 0.2434
Final R indexes [all data]	R_1 = 0.2356, wR_2 = 0.3599
Largest diff. peak/hole	0.61/-0.35 e $Å^{-3}$

References

1. Hikawa, H.; Ino, Y.; Suzuki, H.; Yokoyama, Y. *J. Org. Chem.* **2012,** *77*, 7046-7051. doi: 10.1021/jo301282n.
2. Lin, W.-H.; Wu, W.-C.; Selvaraju, M.; Sun, C.-M. *Org. Chem. Front.* **2017,** *4*, 392-397. doi: 10.1039/C6QO00733C.
3. Laha, J. K.; Satyanarayana Tummalapalli, K. S.; Jethava, K. P. *Org. Biomol. Chem.* **2016,** *14*, 2473-2479. doi: 10.1039/C5OB02670A.
4. Zhan, D.; Li, T.; Zhang, X.; Dai, C.; Wei, H.; Zhang, Y.; Zeng, Q. *Synth. Commun.* **2013,** *43*, 2493-2500. doi: 10.1080/00397911.2012.717669.
5. Lee, J. B.; Kang, M. E.; Kim, J.; Lee, C. Y.; Kee, J.-M.; Myung, K.; Park, J.-U.; Hong, S. Y. *Chem. Commun.* **2017,** *53*, 10394-10397. doi: 10.1039/C7CC05794F.
6. Gupta, P. K.; Hussain, M. K.; Asad, M.; Kant, R.; Mahar, R.; Shukla, S. K.; Hajela, K. *New J. Chem.* **2014,** *38*, 3062-3070. doi: 10.1039/C4NJ00361F.
7. Wu, X.-F.; He, L.; Neumann, H.; Beller, M. *Chem. Eur. J.* **2013,** *19*, 12635-12638. doi: 10.1002/chem.201302182.
8. Mohammed, S.; Vishwakarma, R. A.; Bharate, S. B. *J. Org. Chem.* **2015,** *80*, 6915-6921. doi: 10.1021/acs.joc.5b00989.
9. Rao, K. R.; Mekala, R.; Raghunadh, A.; Meruva, S. B.; Kumar, S. P.; Kalita, D.; Laxminarayana, E.; Prasad, B.; Pal, M. *RSC Adv.* **2015,** *5*, 61575-61579. doi: 10.1039/C5RA10928K.
10. Chen, X.; Chen, T.; Zhou, Y.; Han, D.; Han, L.-B.; Yin, S.-F. *Org. Biomol. Chem.* **2014,** *12*, 3802-3807. doi: 10.1039/C4OB00578C.
11. Parua, S.; Das, S.; Sikari, R.; Sinha, S.; Paul, N. D. *J. Org. Chem.* **2017,** *82*, 7165-7175. doi: 10.1021/acs.joc.7b00643.

12. SAINT+, Bruker AXS Inc., Madison, Wisconsin, USA, 1999 (Program for Reduction of Data collected on Bruker CCD Area Detector Diffractometer V. 6.02.)

13. SADABS, Bruker AXS, Madison, Wisconsin, USA, 2004

14. Sheldrick, G. *Acta Crystallogr. Sect. A* **2008,** *64*, 112-122. doi: doi:10.1107/S0108767307043930.

NMR spectra

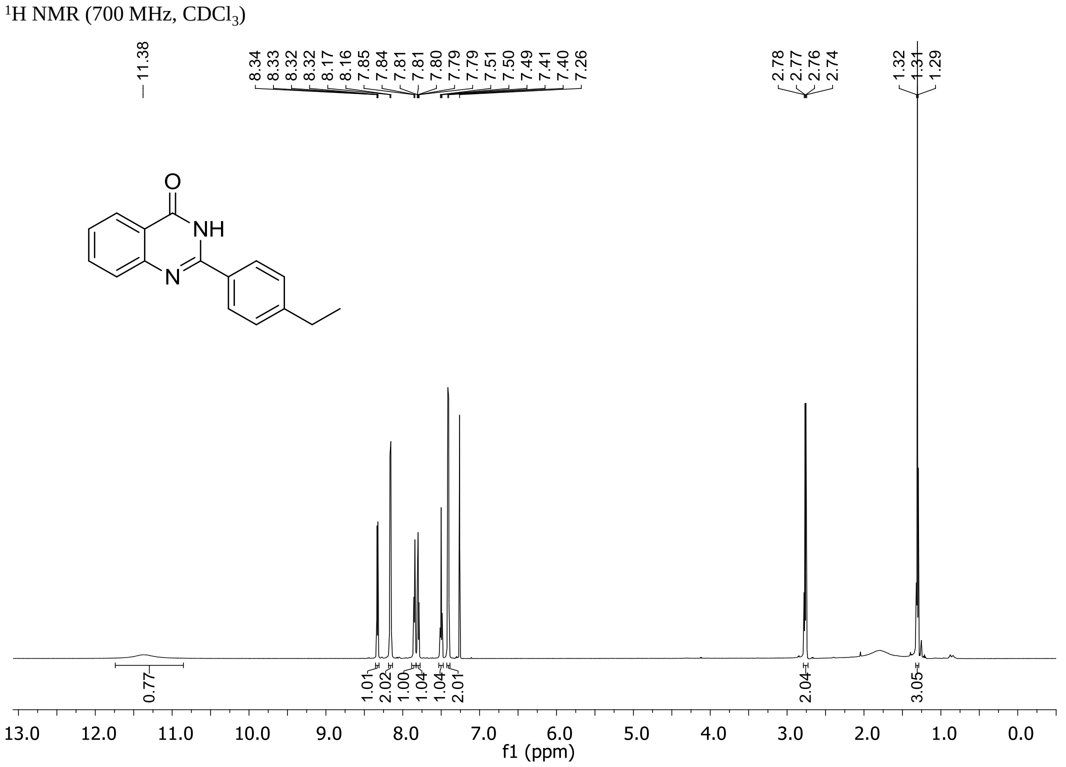

Figure S2. ^{1}H NMR spectra of 2-(4-ethylphenyl)quinazolin-4(3*H*)-one (**3a**).

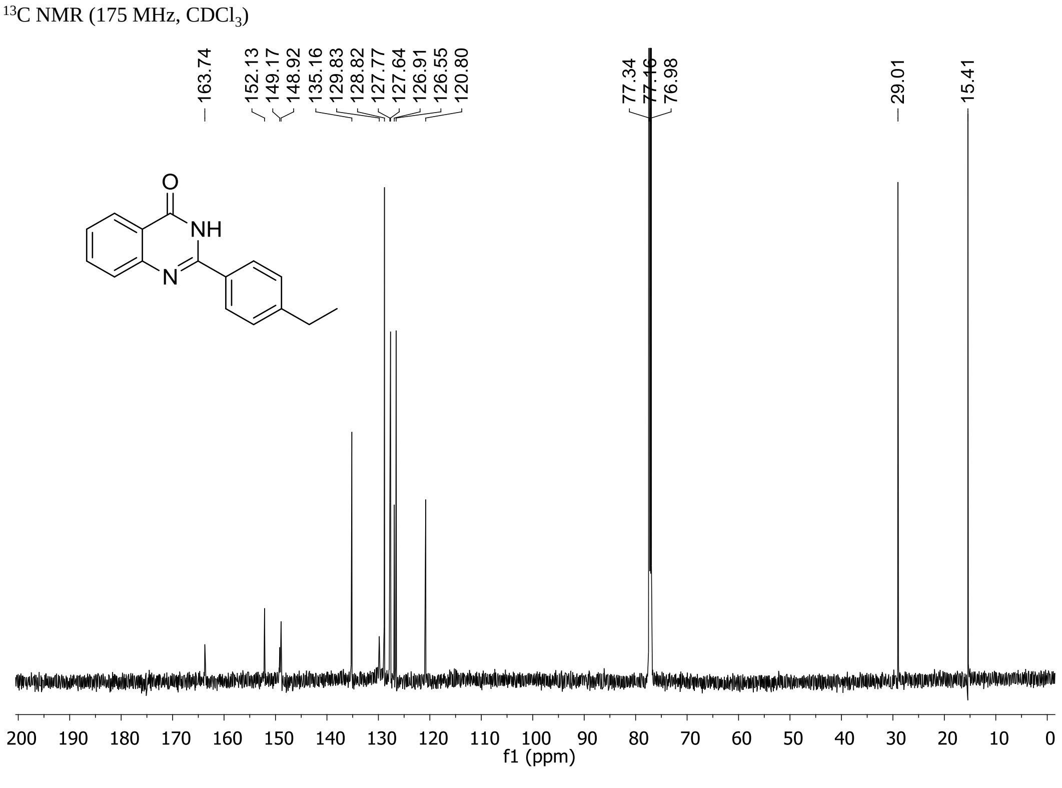

Figure S3. ^{13}C NMR spectra of 2-(4-ethylphenyl)quinazolin-4(3*H*)-one (**3a**).

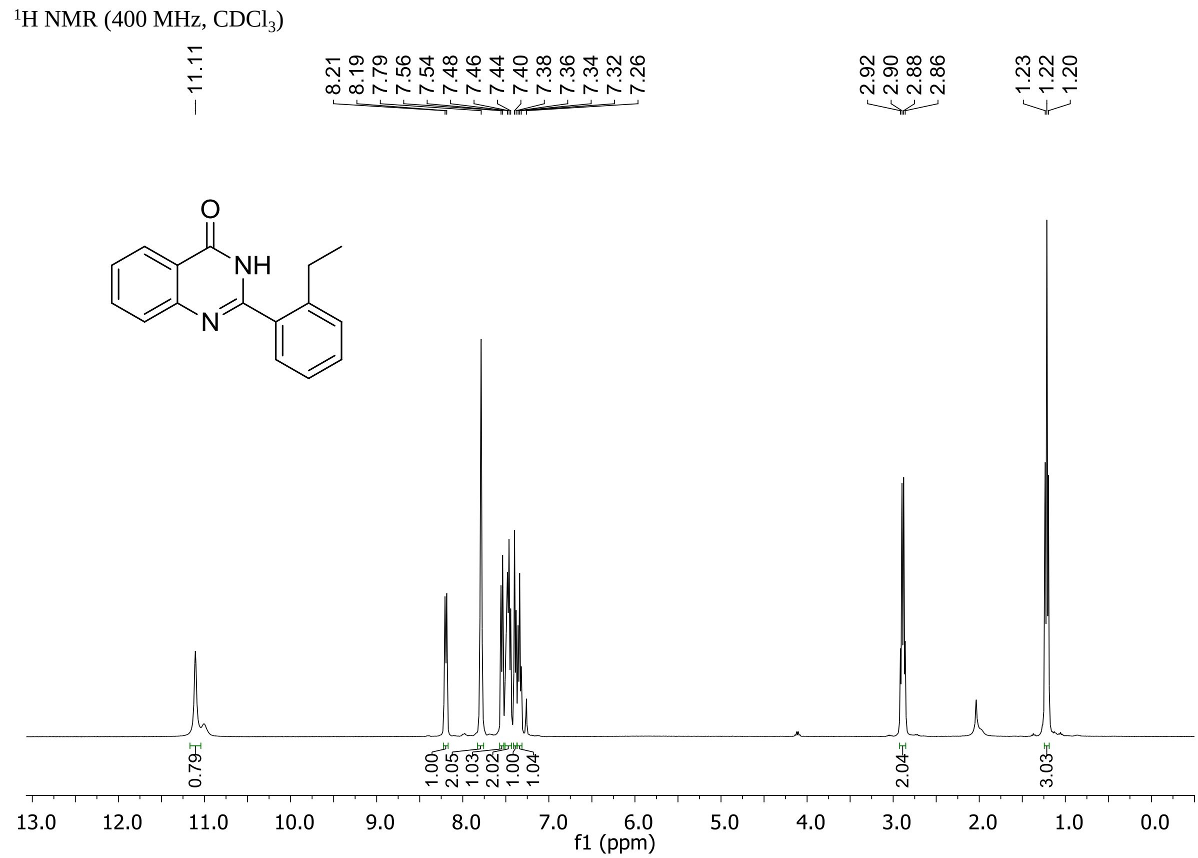

Figure S4. ^{1}H NMR spectra of 2-(2-ethylphenyl)quinazolin-4(3*H*)-one (**3b**).

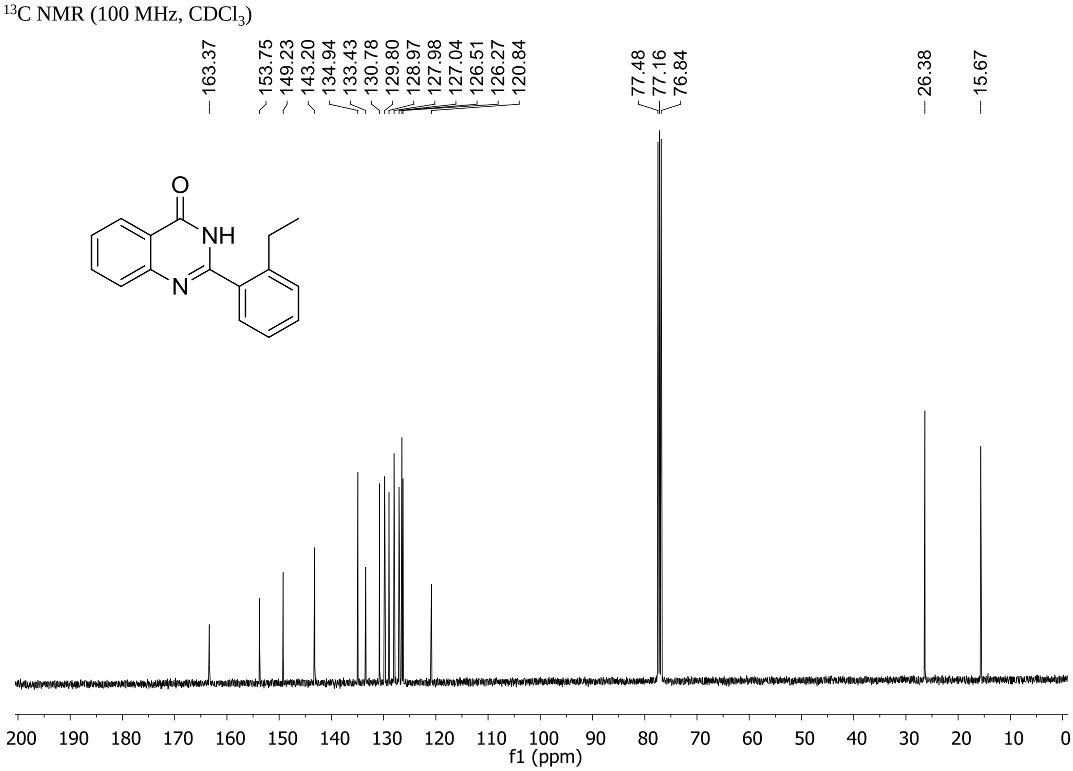

Figure S5. ^{13}C NMR spectra of 2-(2-ethylphenyl)quinazolin-4(3*H*)-one (**3b**)

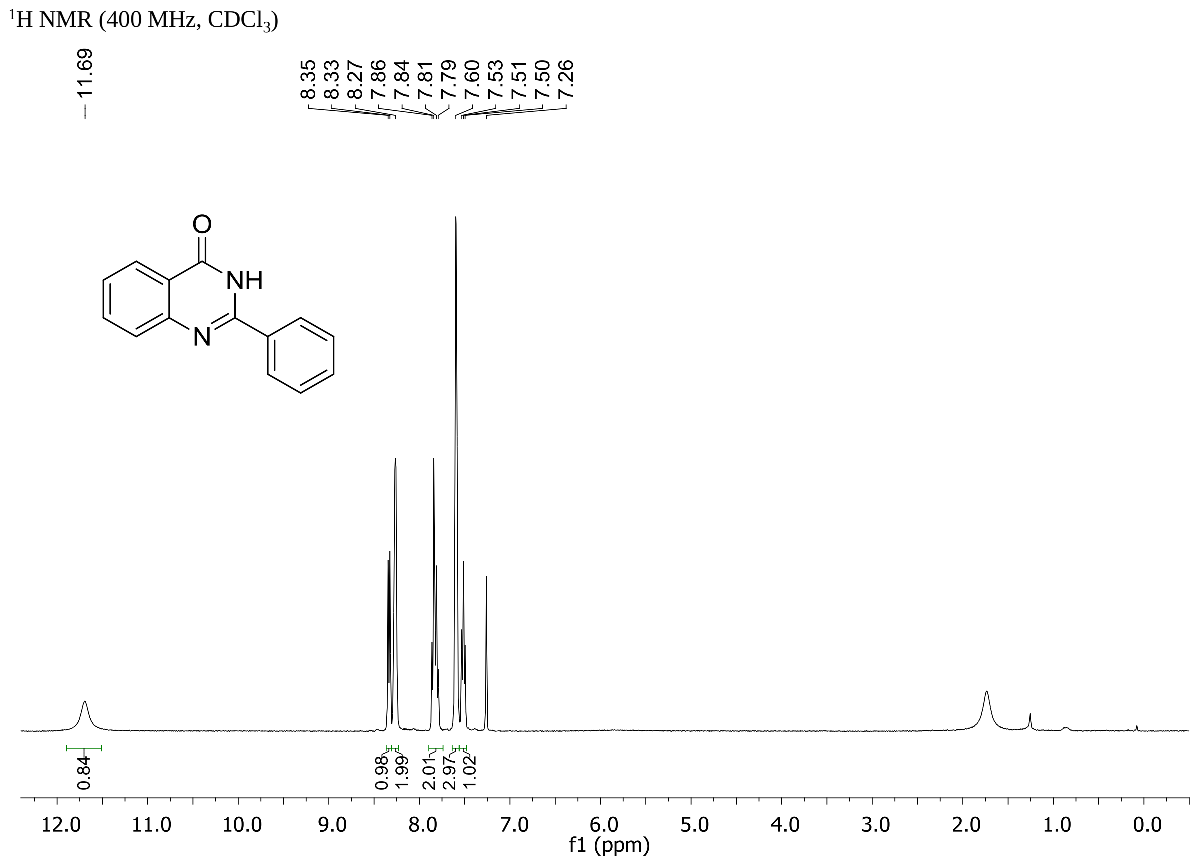

Figure S6. ^{1}H NMR spectra of 2-phenylquinazolin-4(3*H*)-one (**3c**).

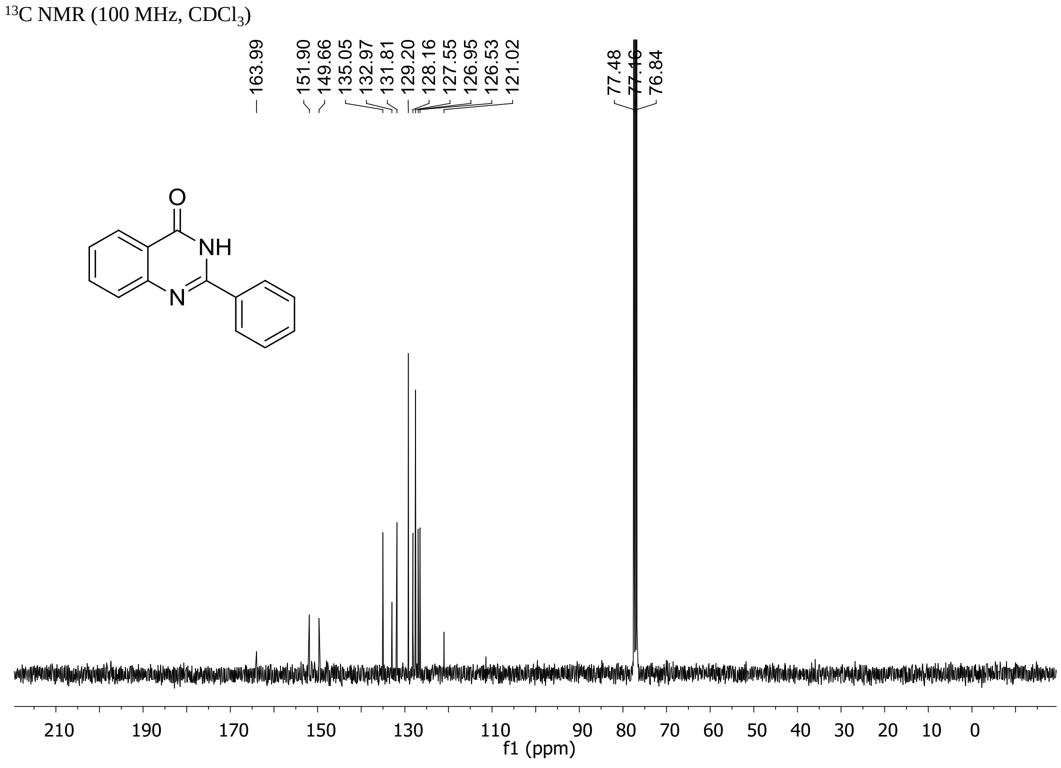

Figure S7. ^{13}C NMR spectra of 2-phenylquinazolin-4(3*H*)-one (**3c**).

^{1}H NMR (400 MHz, $CDCl_3$)

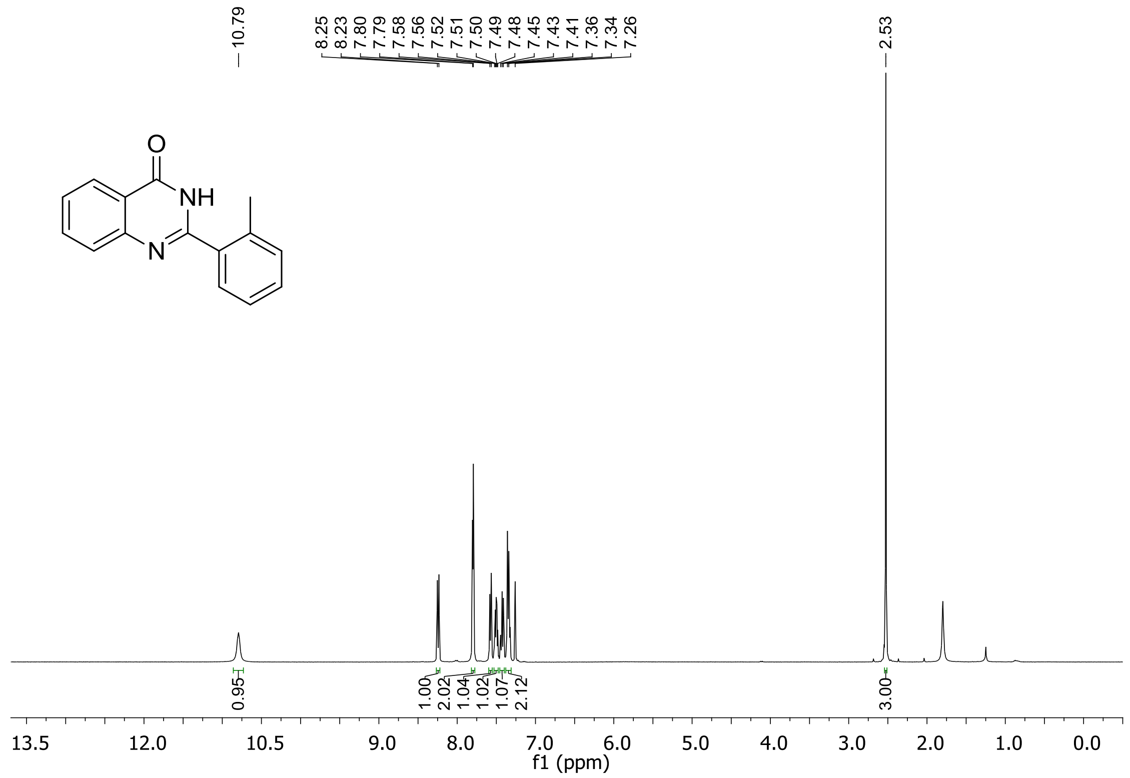

Figure S8. ^{1}H NMR spectra of 2-(*o*-tolyl)quinazolin-4(3*H*)-one (**3d**).

^{13}C NMR (100 MHz, $CDCl_3$)

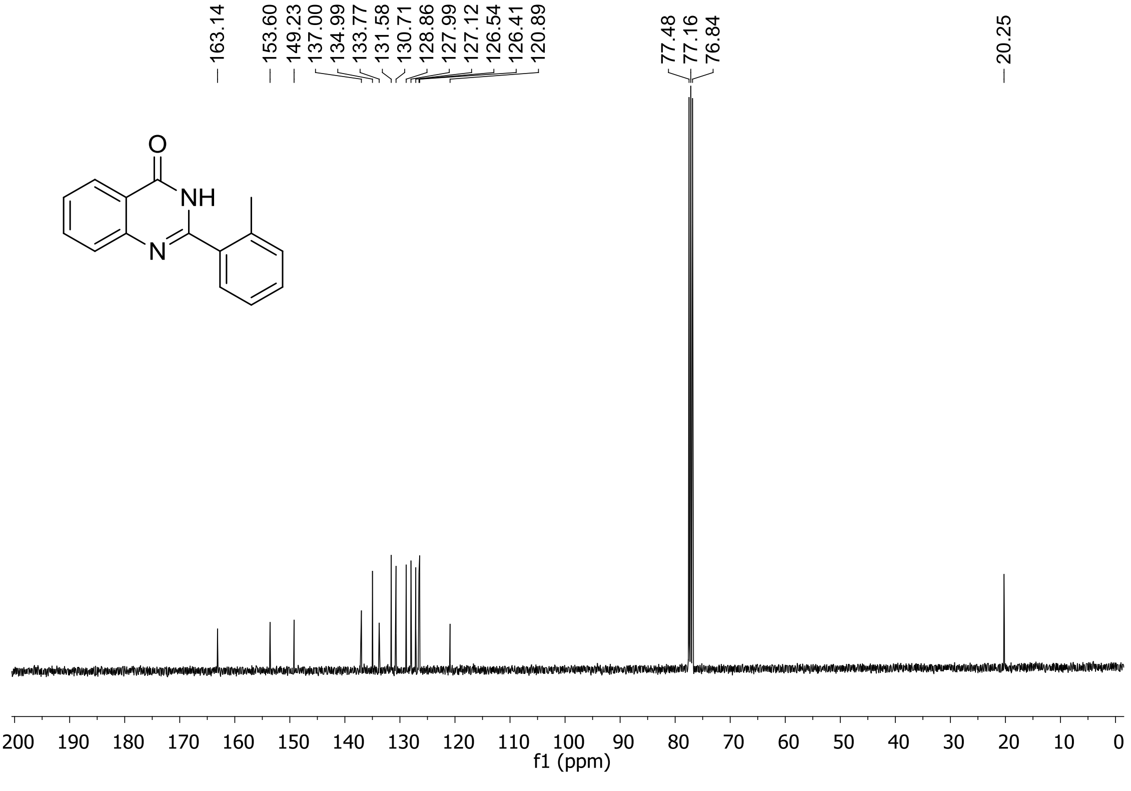

Figure S9. ^{13}C NMR spectra of 2-(*o*-tolyl)quinazolin-4(3*H*)-one (**3d**).

^{1}H NMR (700 MHz, DMSO-d_6)

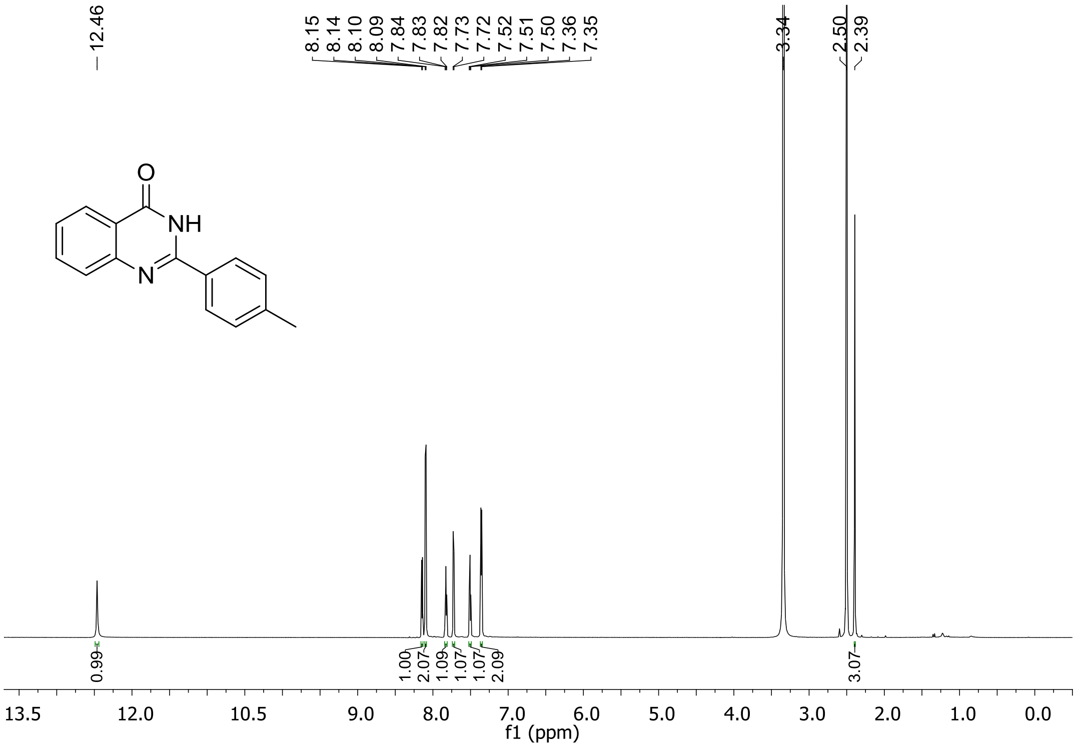

Figure S10. ^{1}H NMR spectra of 2-(*p*-tolyl)quinazolin-4(3*H*)-one (**3e**).

^{1}H NMR (175 MHz, DMSO-d_6)

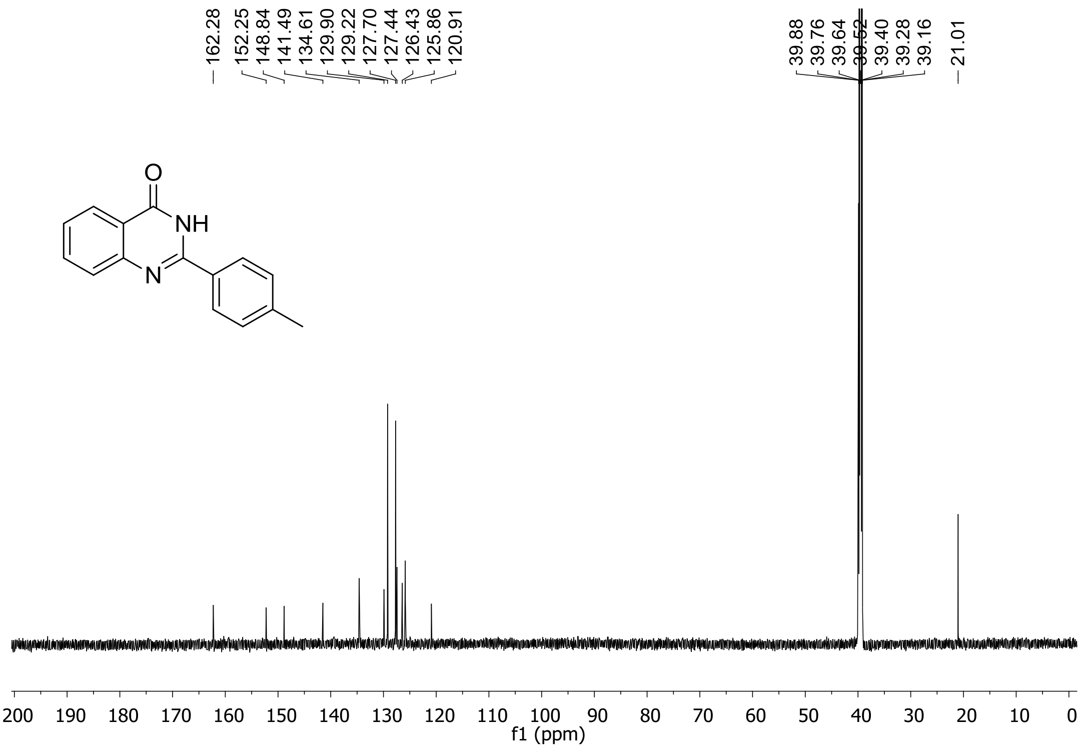

Figure S11. ^{13}C NMR spectra of 2-(*p*-tolyl)quinazolin-4(3*H*)-one (**3e**).

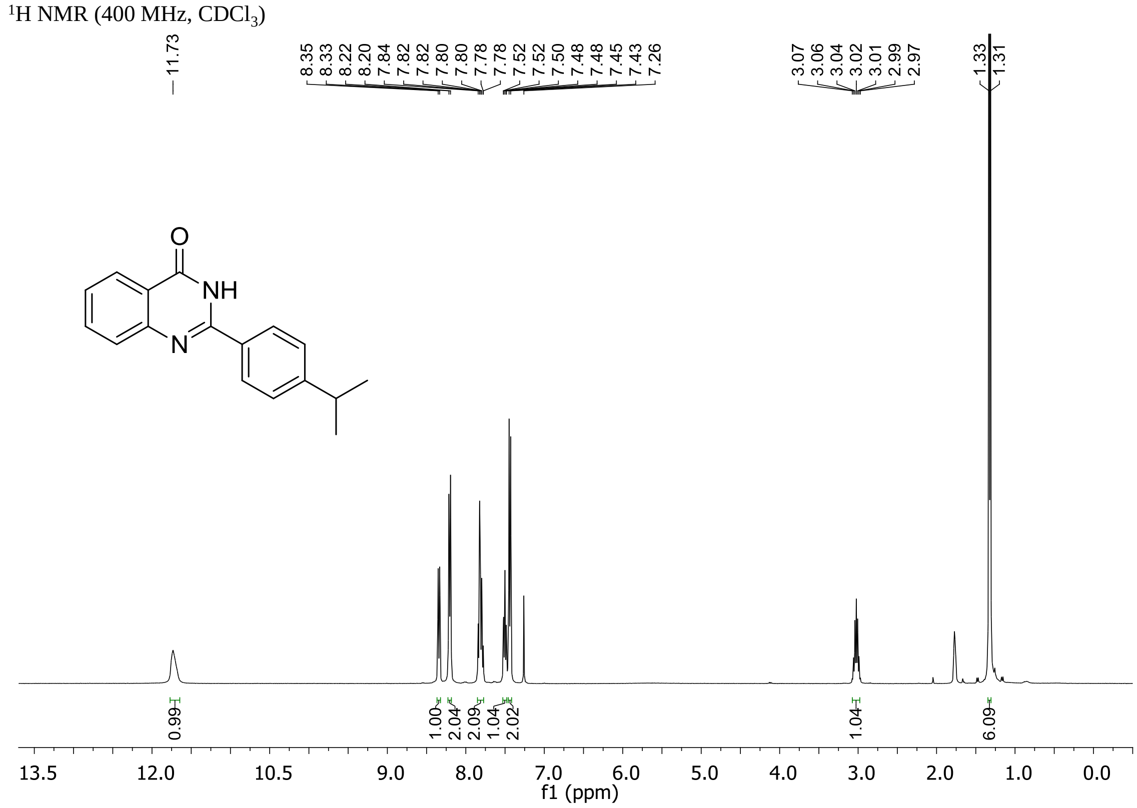

Figure S12. ^{1}H NMR spectra of 2-(4-isopropylphenyl)quinazolin-4(3*H*)-one (**3f**).

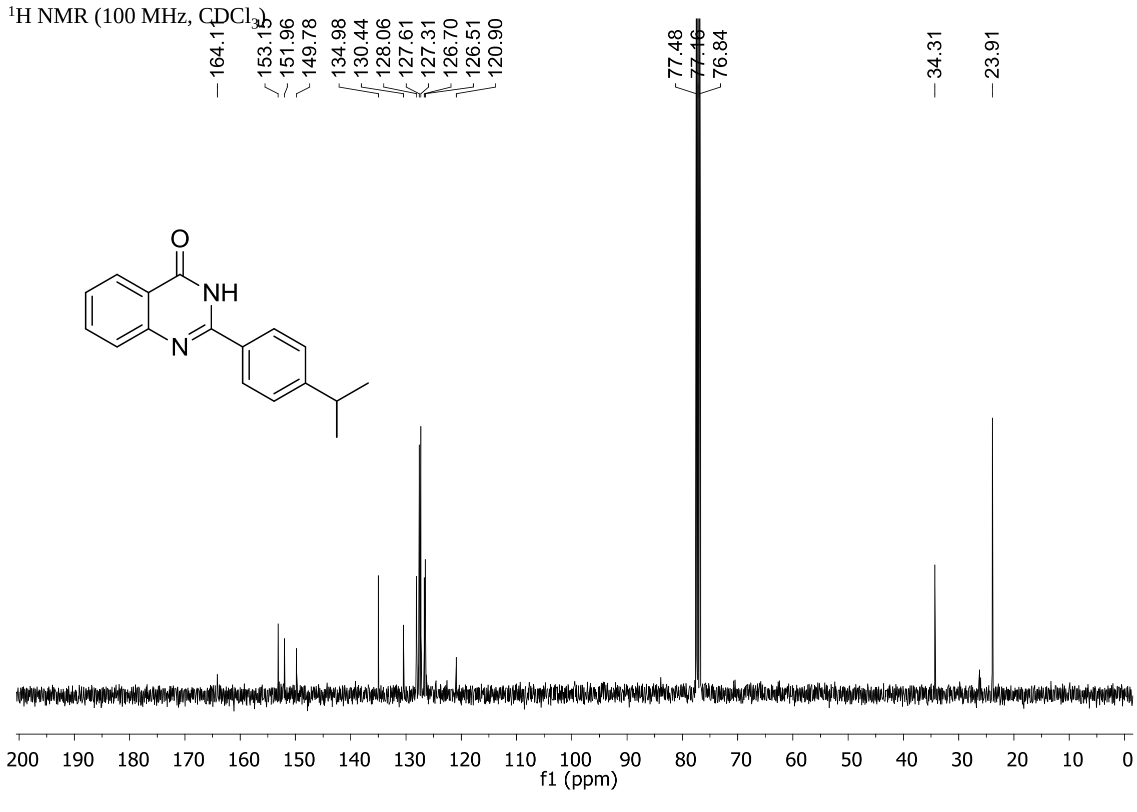

Figure S13. ^{13}C NMR spectra of 2-(4-isopropylphenyl)quinazolin-4(3*H*)-one (**3f**).

^{1}H NMR (400 MHz, DMSO-d6)

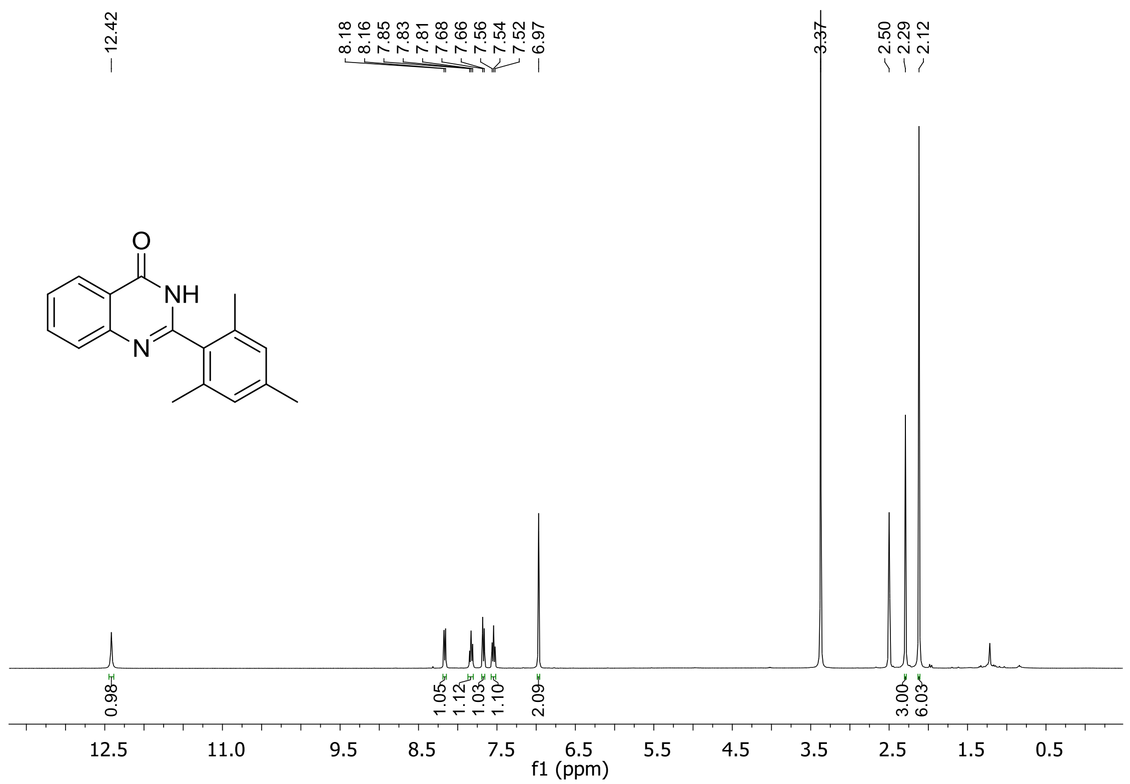

Figure S14. ^{1}H NMR spectra of 2-mesitylquinazolin-4(3*H*)-one (**3g**).

^{13}C NMR (100 MHz, DMSO-d6)

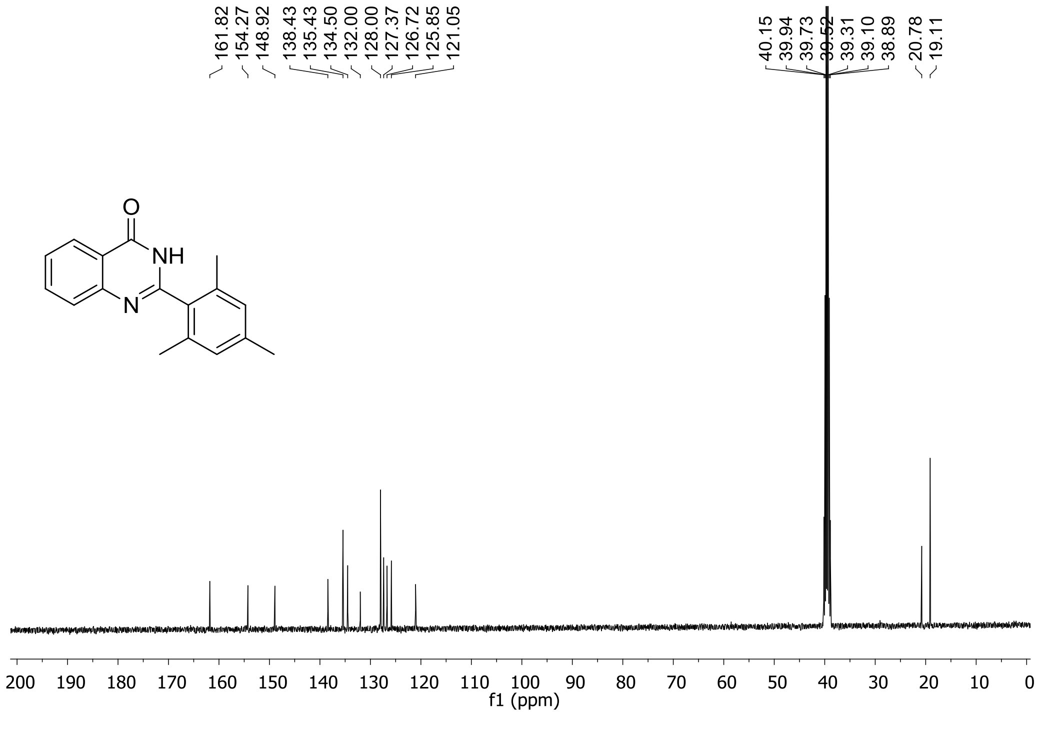

Figure S15. ^{13}C NMR spectra of 2-mesitylquinazolin-4(3*H*)-one (**3g**).

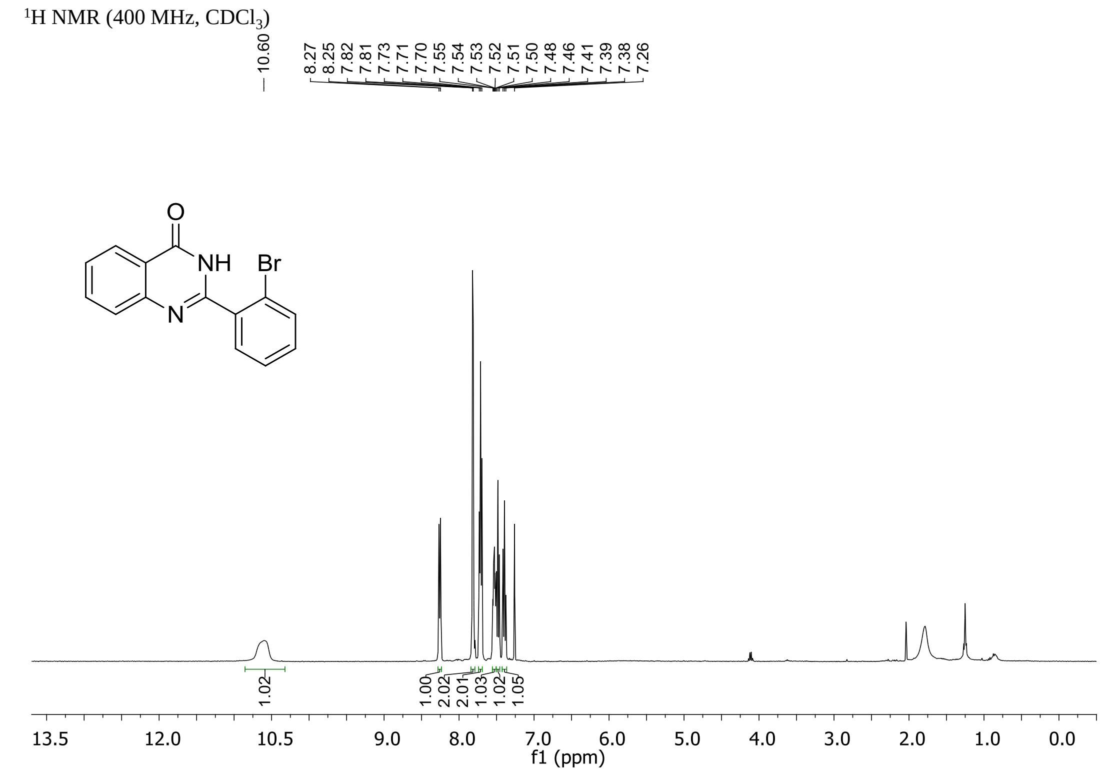

Figure S16. ^{1}H NMR spectra of 2-(2-bromophenyl)quinazolin-4(3*H*)-one (**3h**).

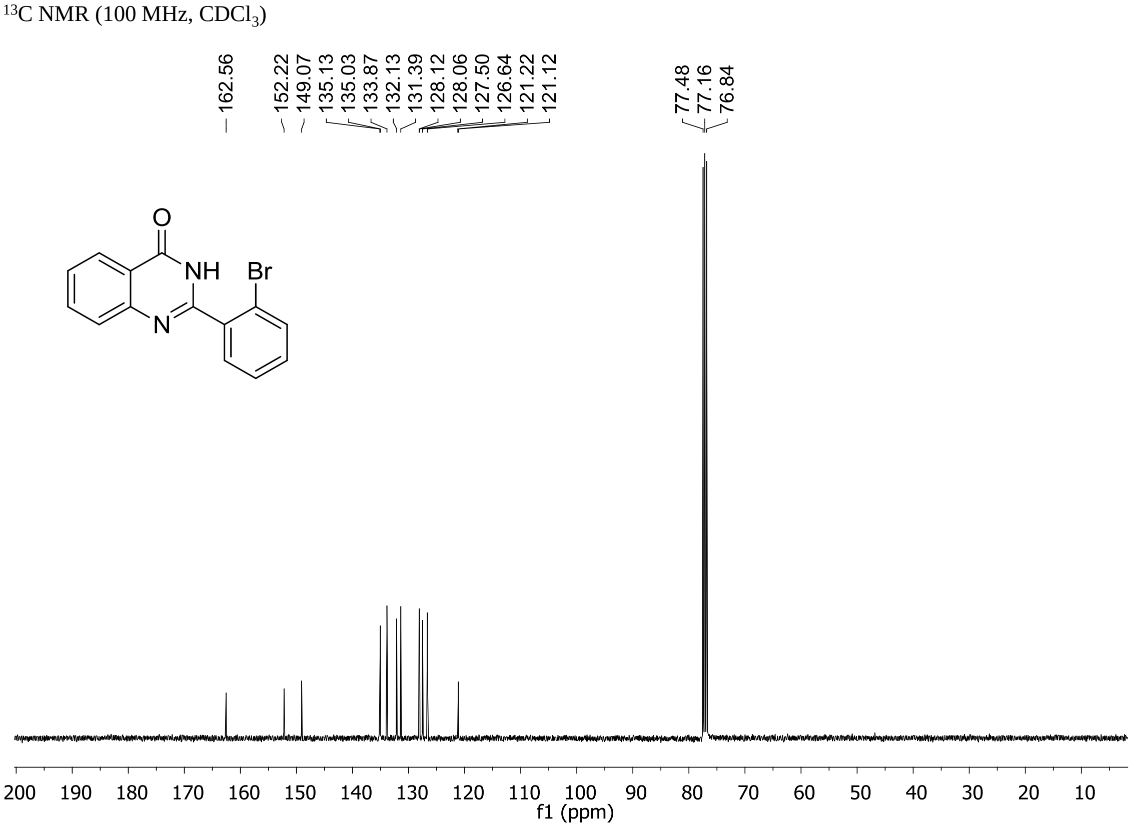

Figure S17. ^{13}C NMR spectra of 2-(2-bromophenyl)quinazolin-4(3*H*)-one (**3h**).

^{1}H NMR (400 MHz, $CDCl_3$)

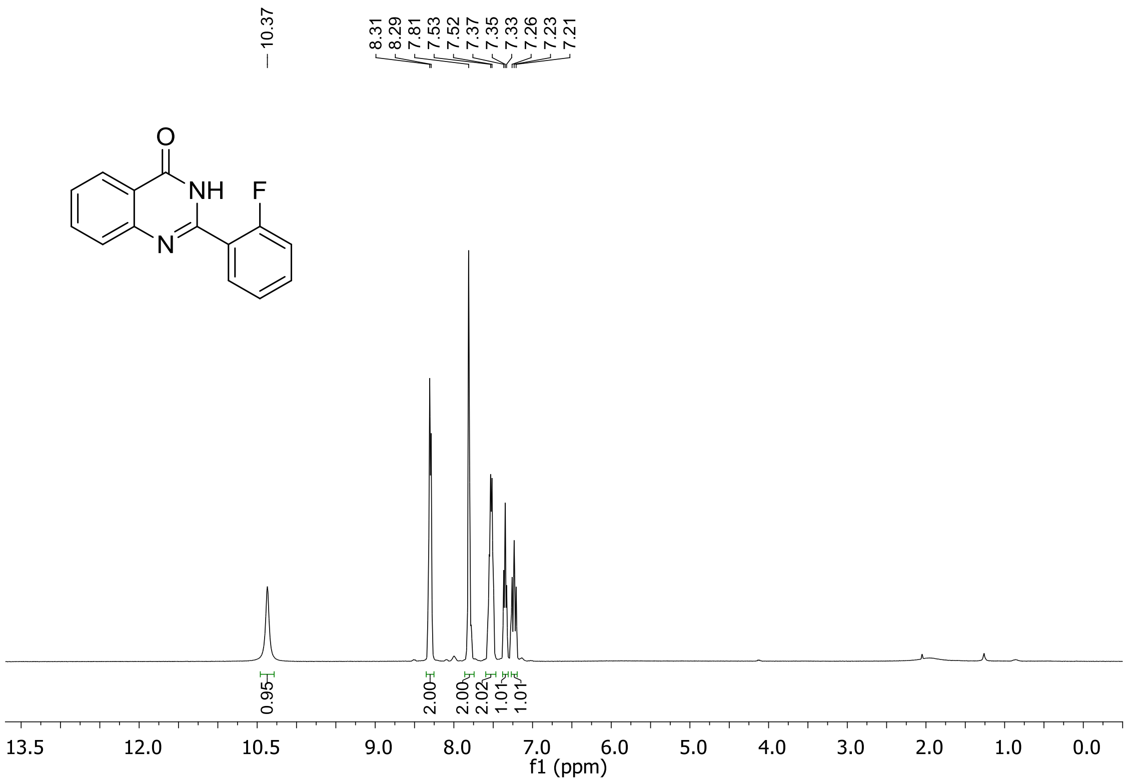

Figure S18. ^{1}H NMR spectra of 2-(2-fluorophenyl)quinazolin-4(3*H*)-one (**3i**).

^{13}C NMR (100 MHz, $CDCl_3$)

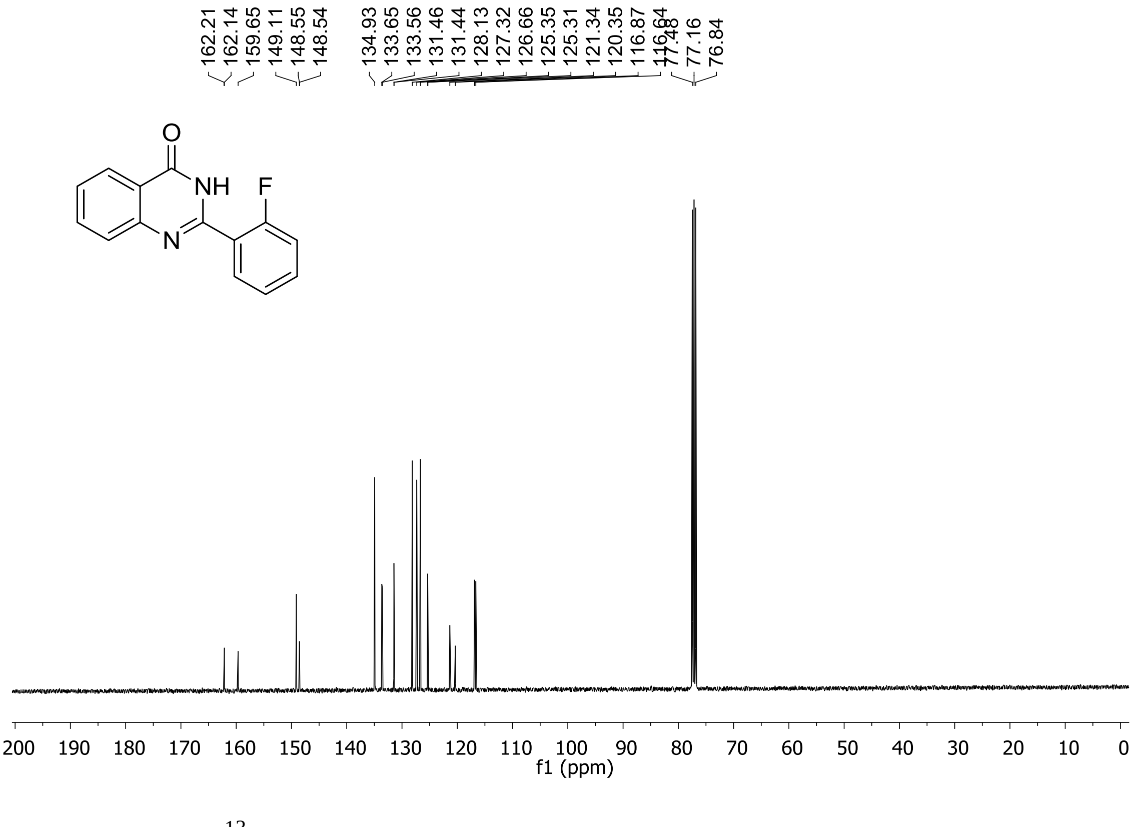

Figure S19. ^{13}C NMR spectra of 2-(2-fluorophenyl)quinazolin-4(3*H*)-one (**3i**).

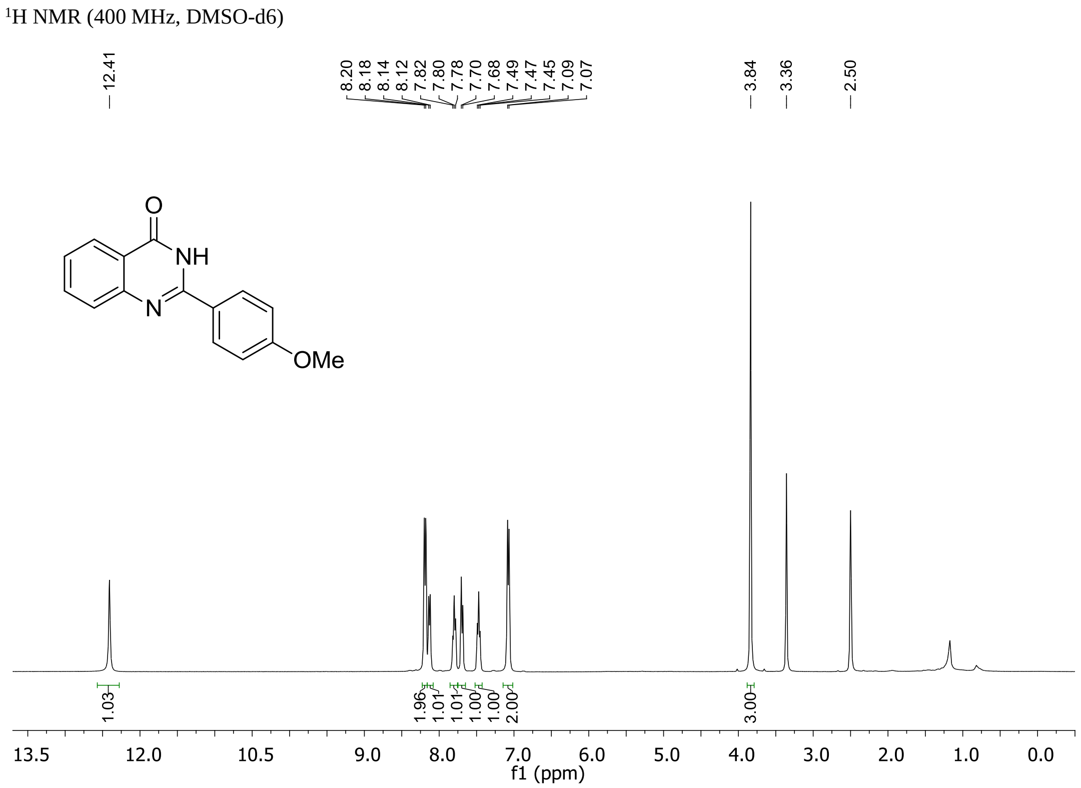

Figure S20. ^{1}H NMR spectra of 2-(4-methoxyphenyl)quinazolin-4(3*H*)-one (**3j**).

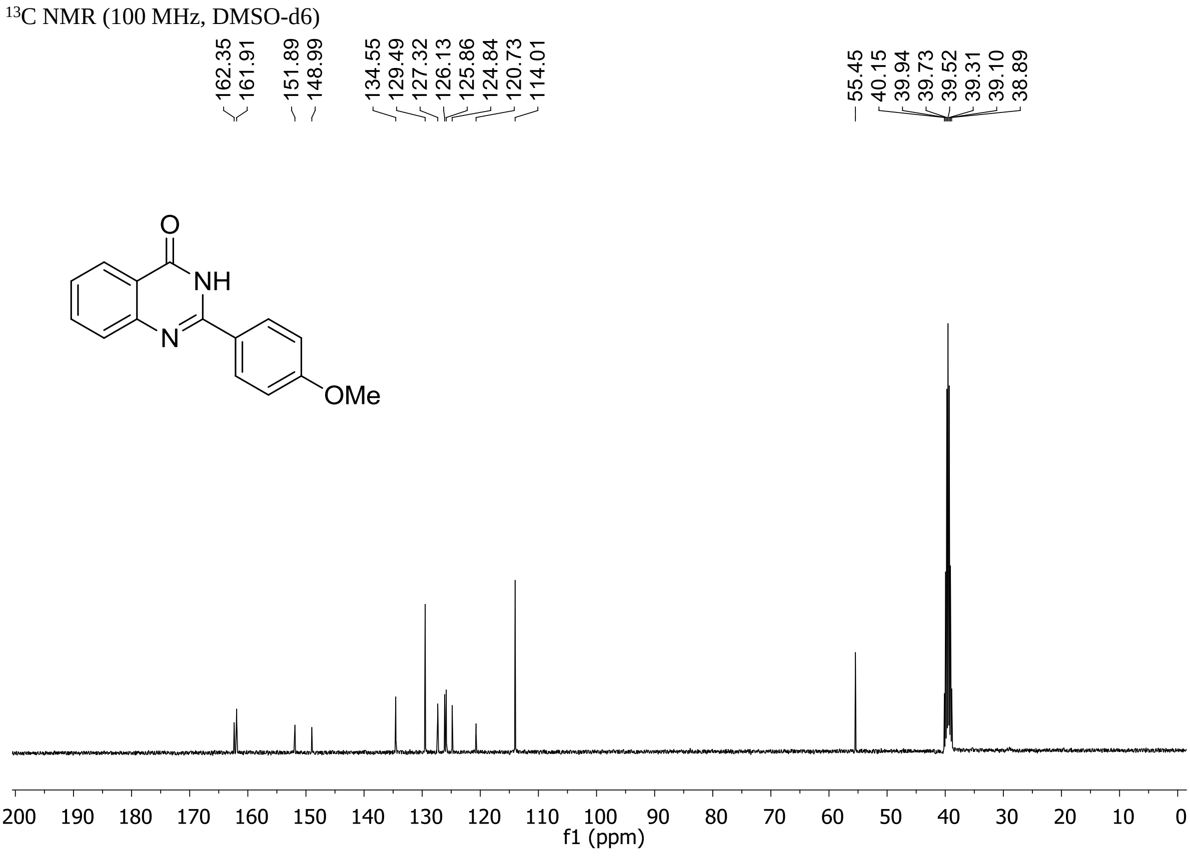

Figure S21. ^{13}C NMR spectra of 2-(4-methoxyphenyl)quinazolin-4(3*H*)-one (**3j**).

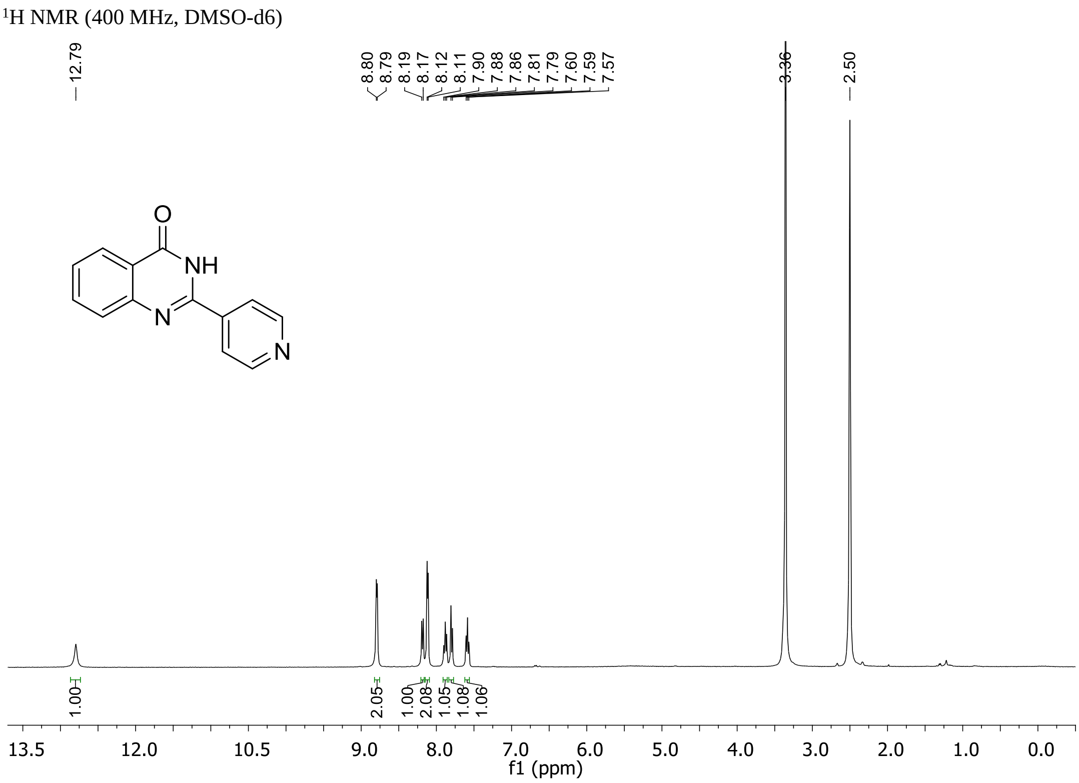

Figure S22. ^{1}H NMR spectra of 2-(pyridin-4-yl)quinazolin-4(3*H*)-one (**3k**).

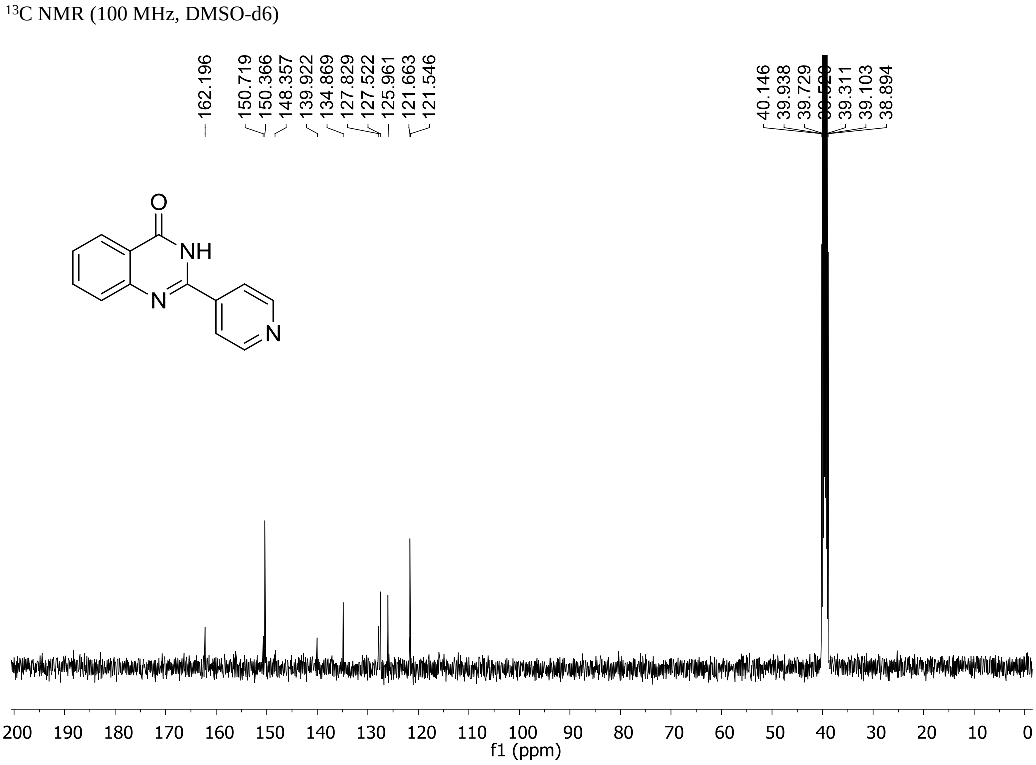

Figure S23. ^{13}C NMR spectra of 2-(pyridin-4-yl)quinazolin-4(3*H*)-one (**3k**).

^{1}H NMR (400 MHz, DMSO-d6)

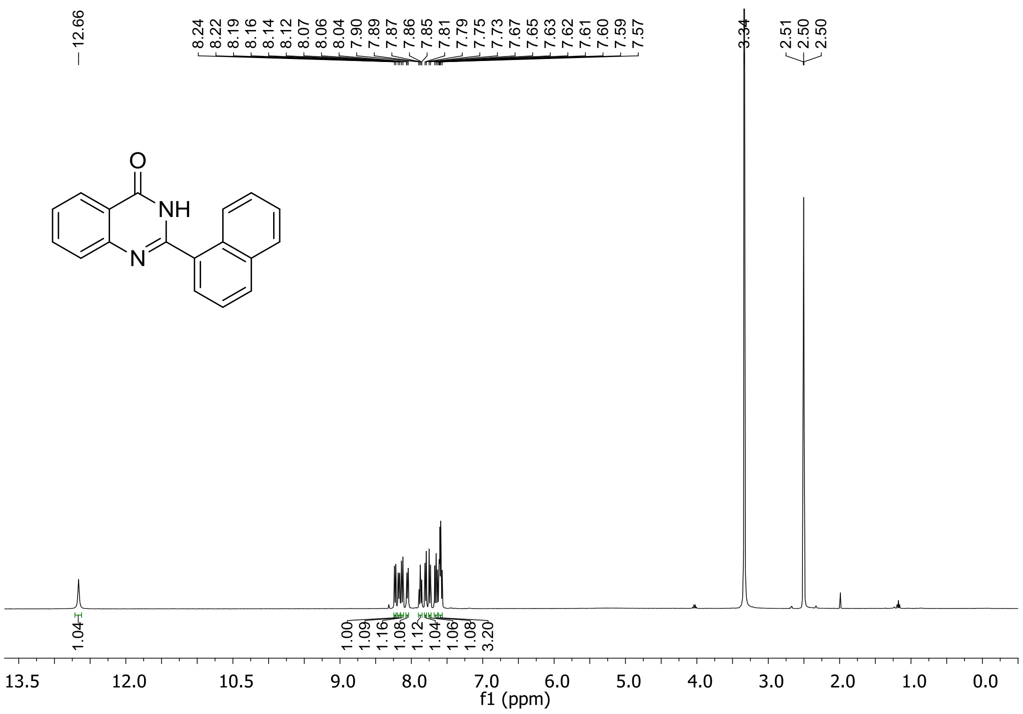

Figure S24. ^{1}H NMR spectra of 2-(naphthalen-1-yl)quinazolin-4(3*H*)-one (**3l**).

^{13}C NMR (100 MHz, DMSO-d6)

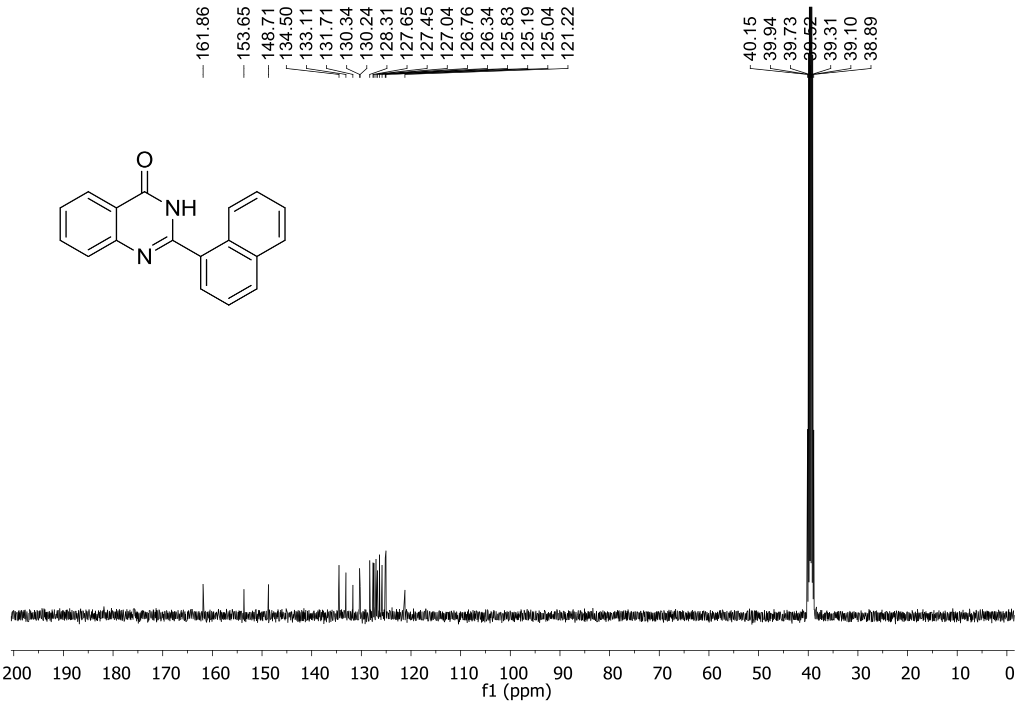

Figure S25. ^{13}C NMR spectra of 2-(naphthalen-1-yl)quinazolin-4(3*H*)-one (**3l**).

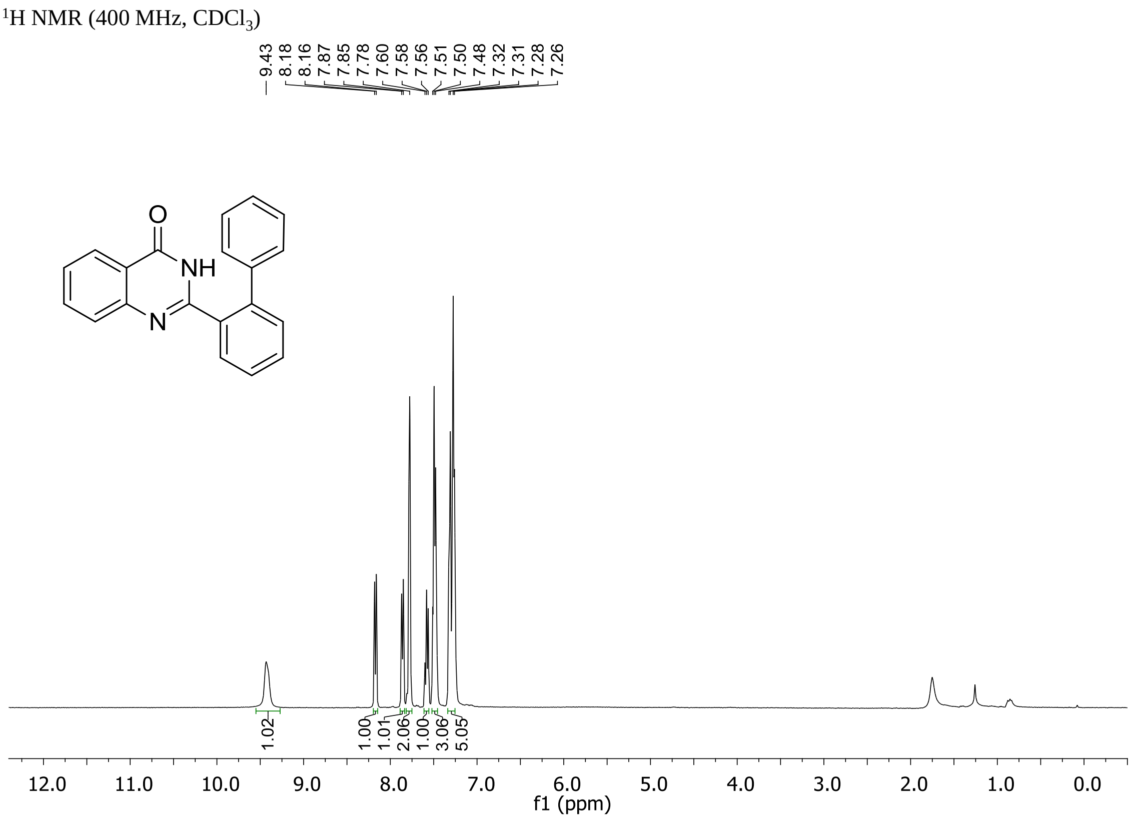

Figure S26. ^{1}H NMR spectra of 2-([1,1'-biphenyl]-2-yl)quinazolin-4(3*H*)-one (**3m**).

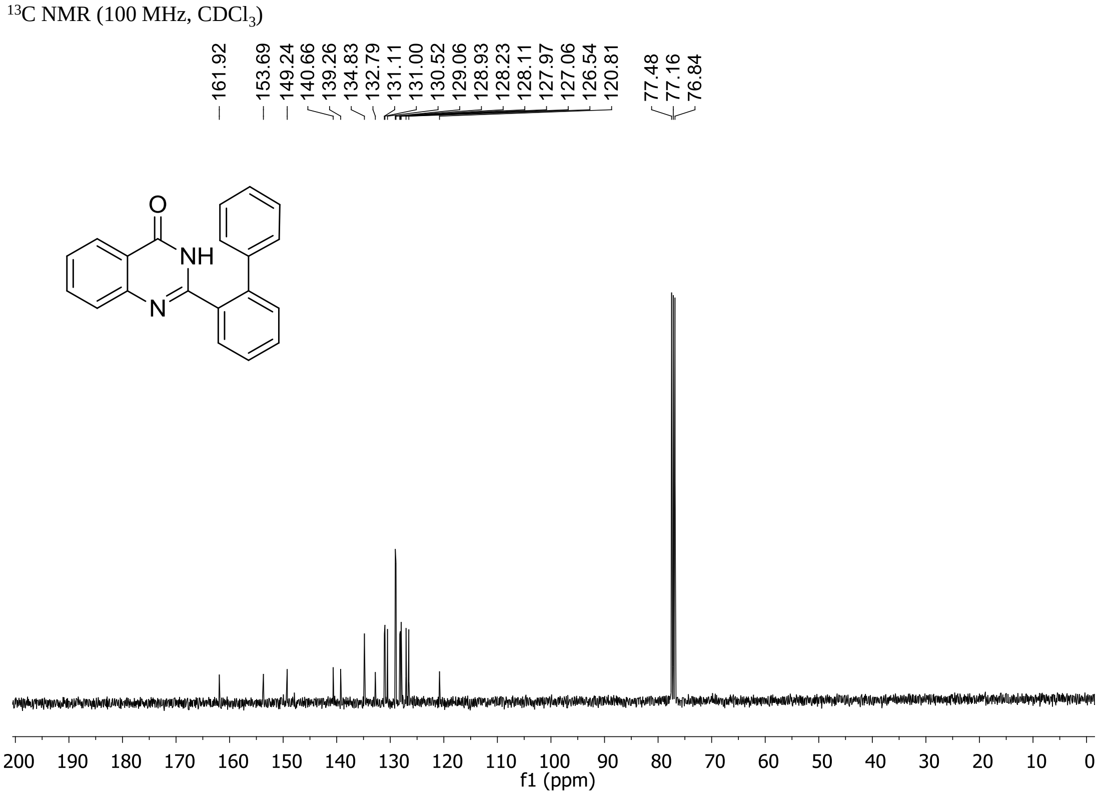

Figure S27. ^{13}C NMR spectra of 2-([1,1'-biphenyl]-2-yl)quinazolin-4(3*H*)-one (**3m**).

^{1}H NMR (400 MHz, $CDCl_3$)

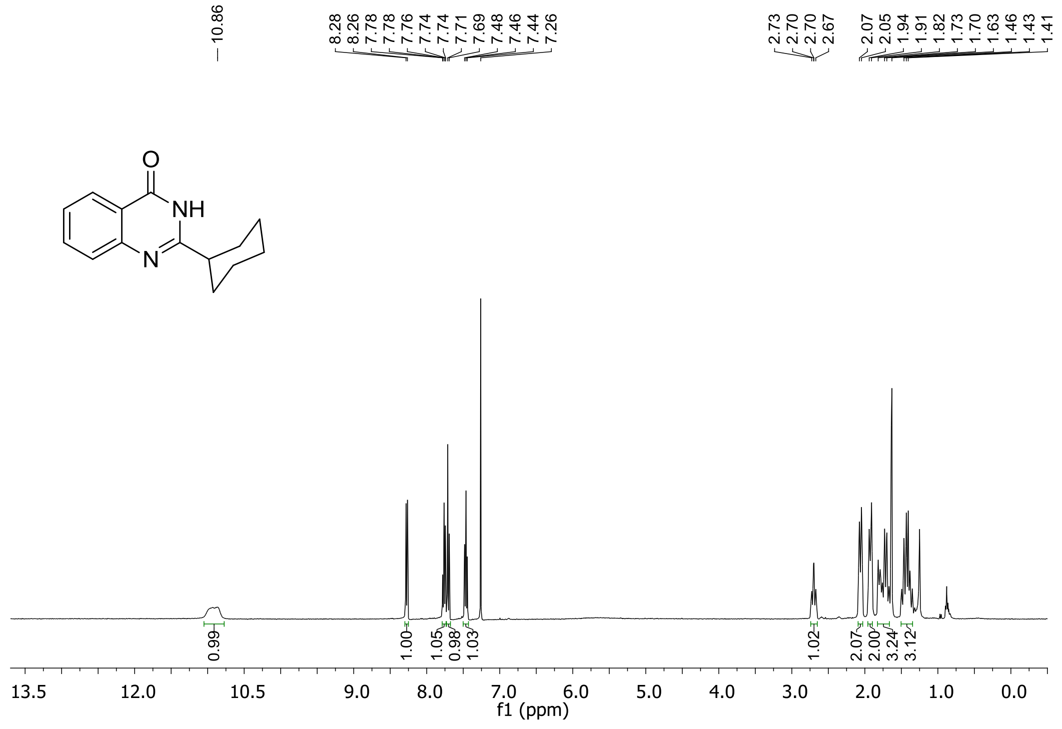

Figure S28. ^{1}H NMR spectra of 2-cyclohexylquinazolin-4(3*H*)-one (**3n**).

^{13}C NMR (100 MHz, $CDCl_3$)

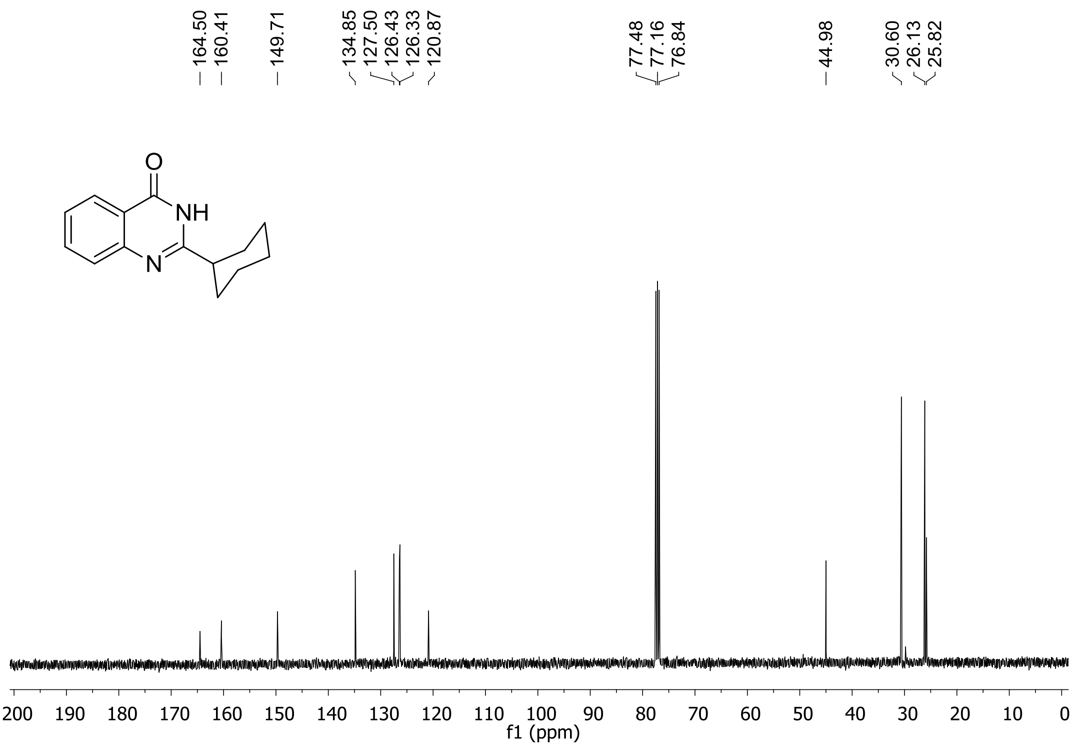

Figure S29. ^{13}C NMR spectra of 2-cyclohexylquinazolin-4(3*H*)-one (**3n**).

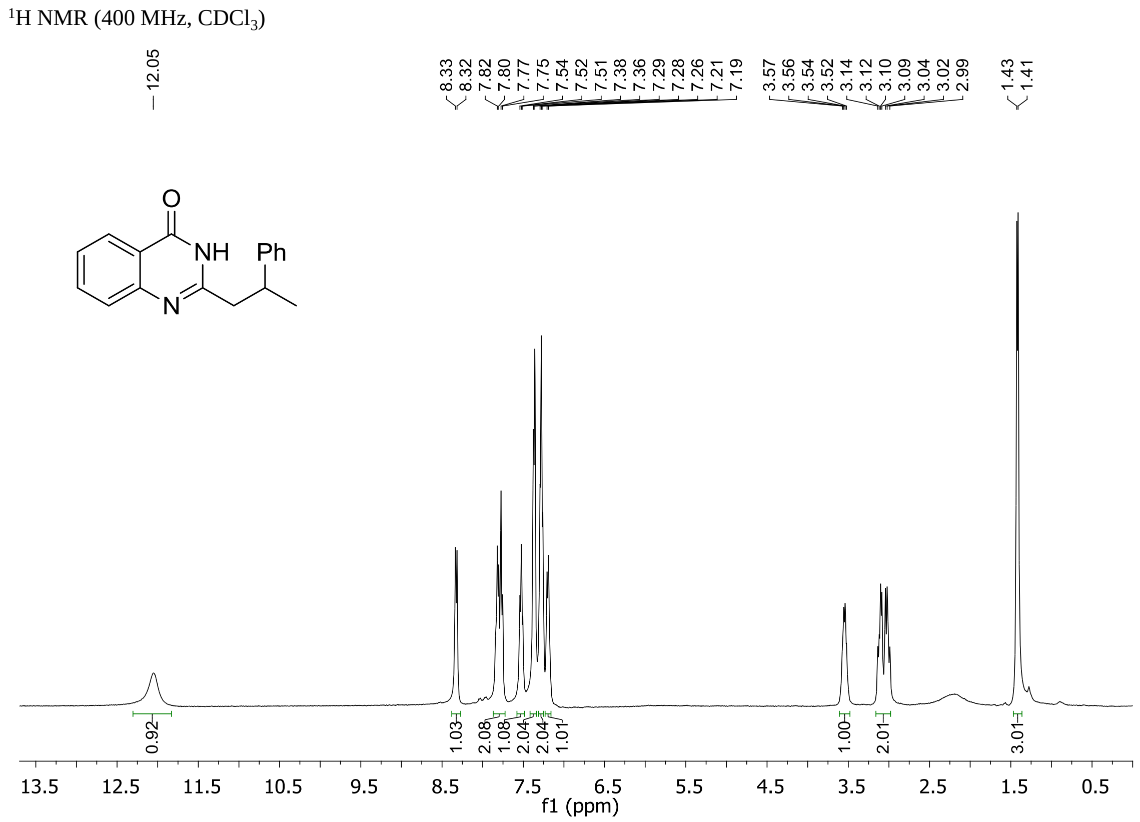

Figure S30. ^{1}H NMR spectra of 2-(2-phenylpropyl)quinazolin-4(3*H*)-one (**3o**).

.

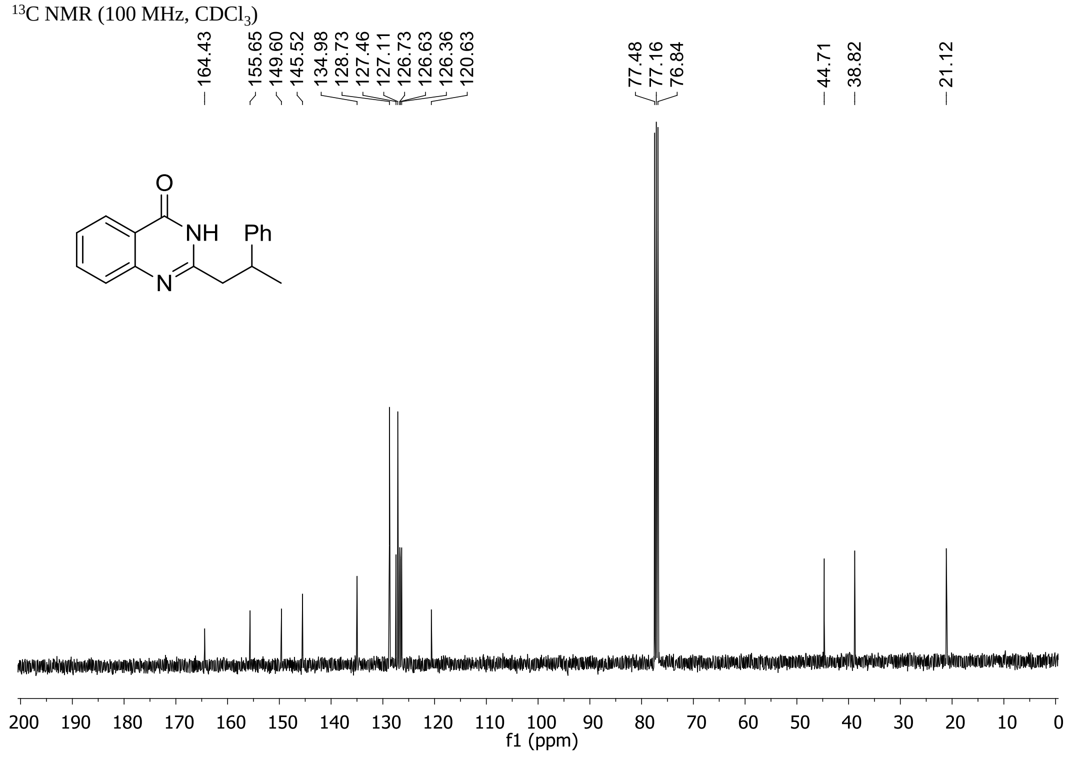

Figure S31. ^{13}C NMR spectra of 2-(2-phenylpropyl)quinazolin-4(3*H*)-one (**3o**).

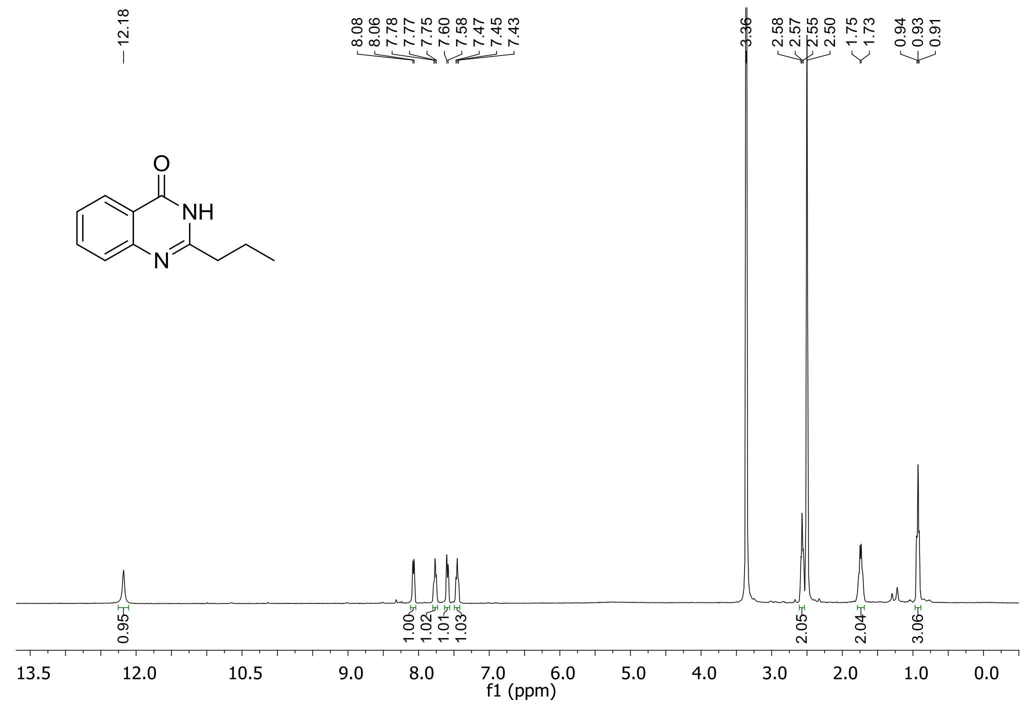

Figure S32. ^{1}H NMR spectra of 2-propylquinazolin-4(3*H*)-one (**3p**).

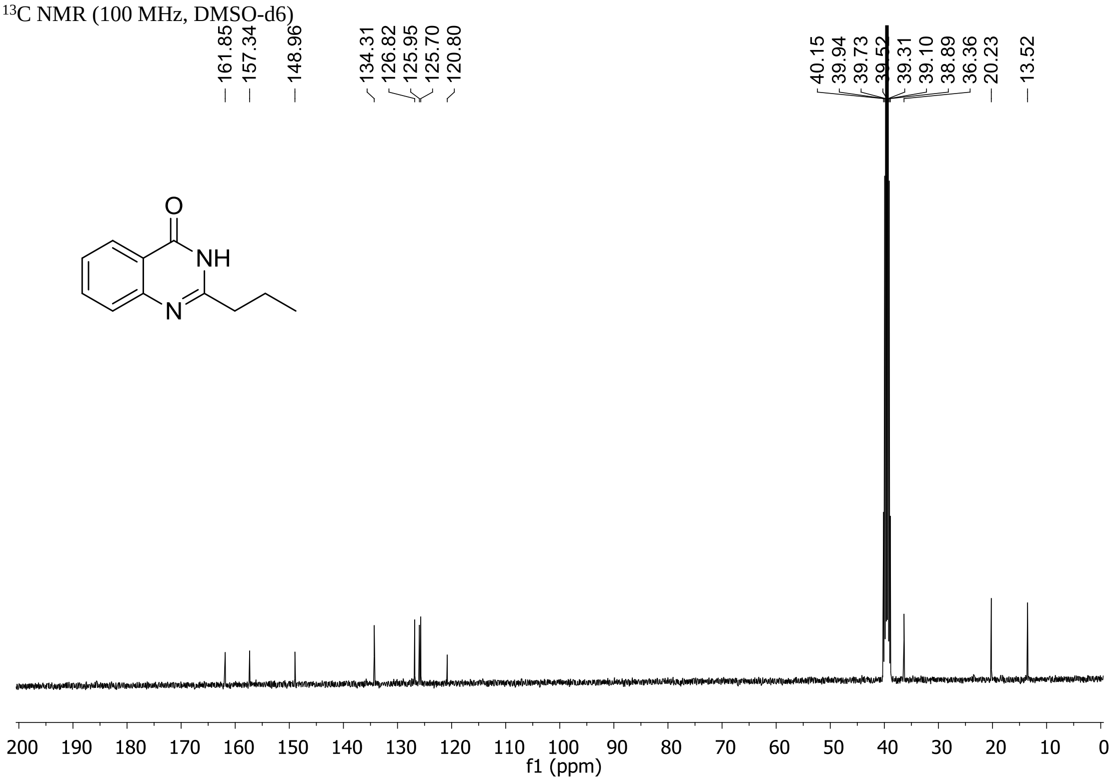

Figure S33. ^{13}C NMR spectra of 2-propylquinazolin-4(3*H*)-one (**3p**).

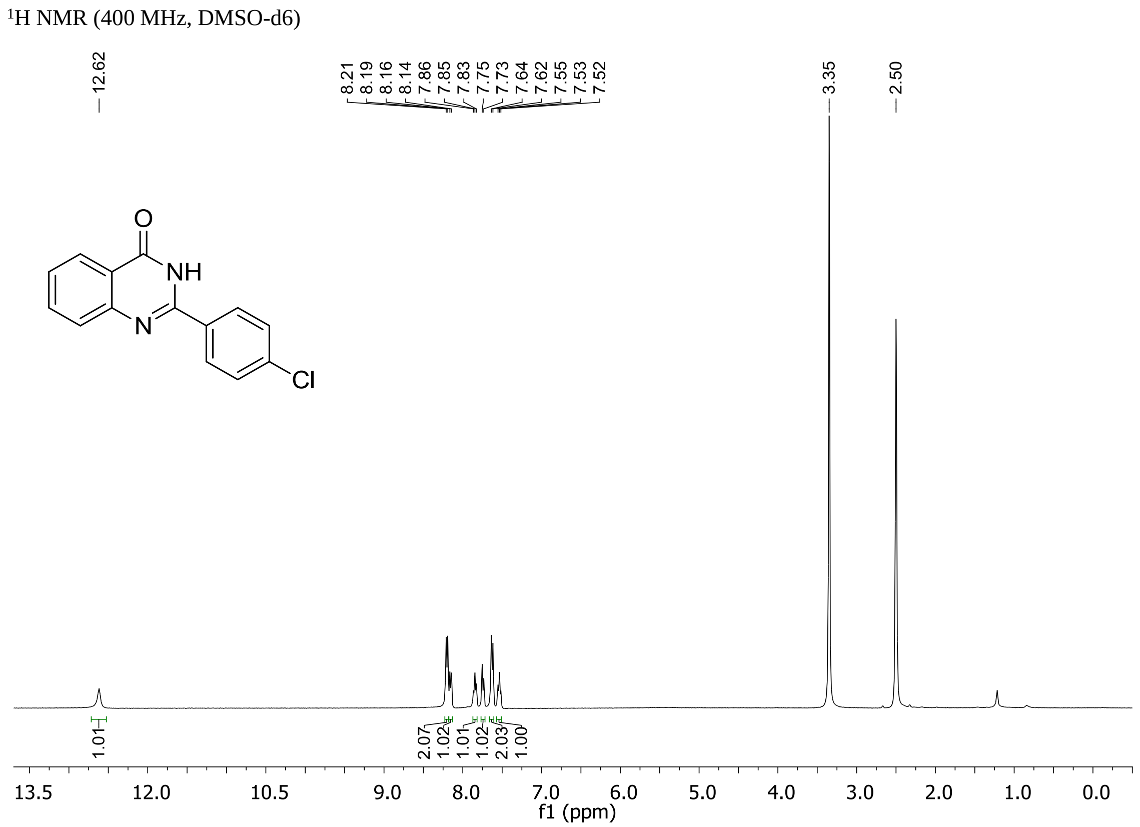

Figure S34. ^{1}H NMR spectra of 2-(4-chlorophenyl)quinazolin-4(3*H*)-one (**3q**).

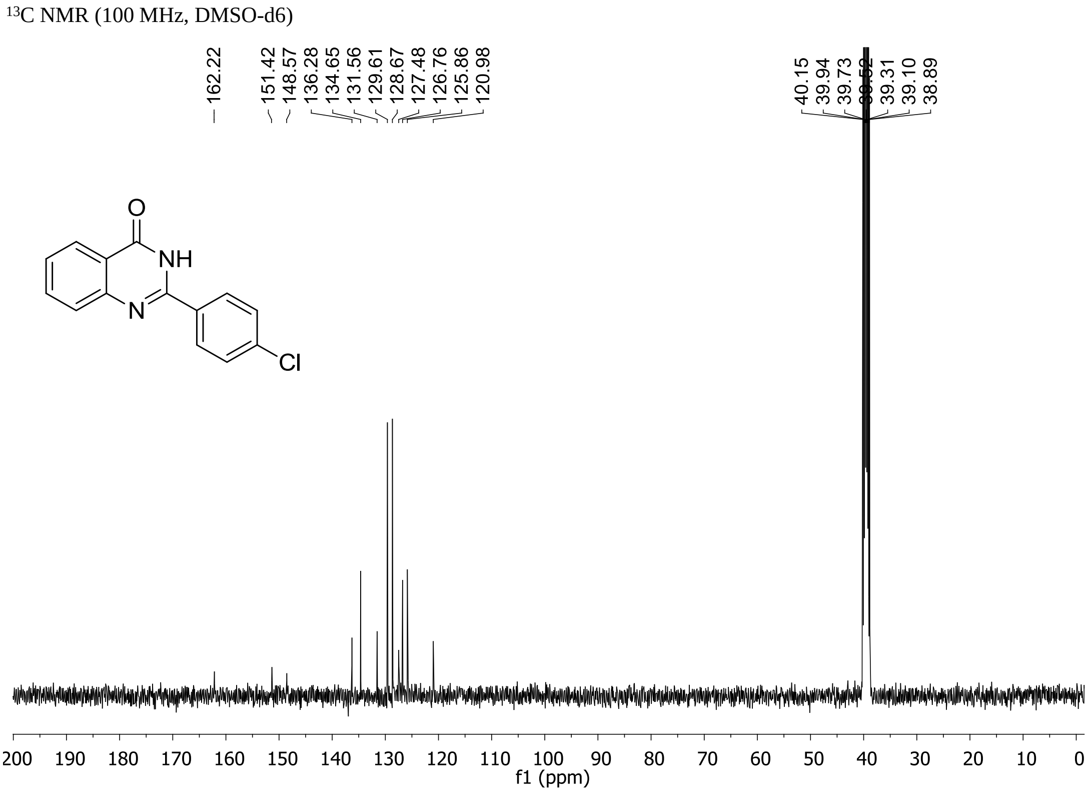

Figure S35. ^{13}C NMR spectra of 2-(4-chlorophenyl)quinazolin-4(3*H*)-one (**3q**).

^{1}H NMR (400 MHz, DMSO-d6)

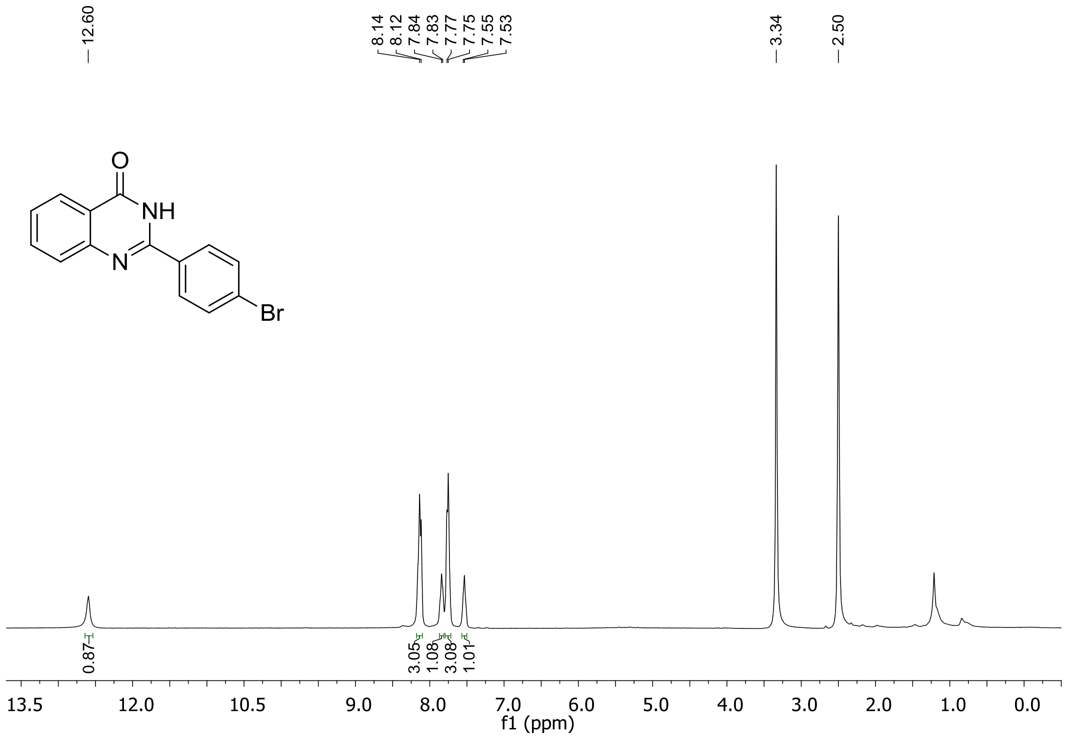

Figure S36. ^{1}H NMR spectra of 2-(4-bromophenyl)quinazolin-4(3*H*)-one (**3r**).

^{13}C NMR (175 MHz, DMSO-d6)

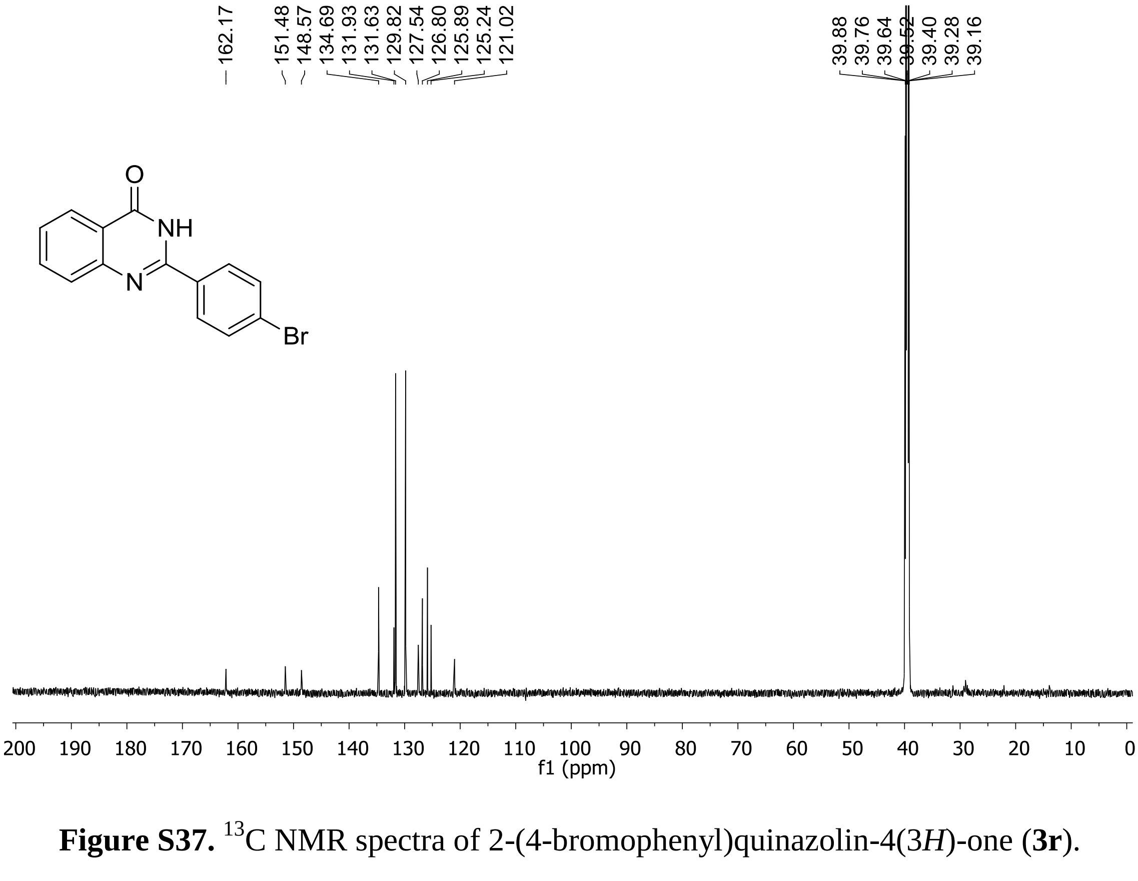

Figure S37. ^{13}C NMR spectra of 2-(4-bromophenyl)quinazolin-4(3*H*)-one (**3r**).

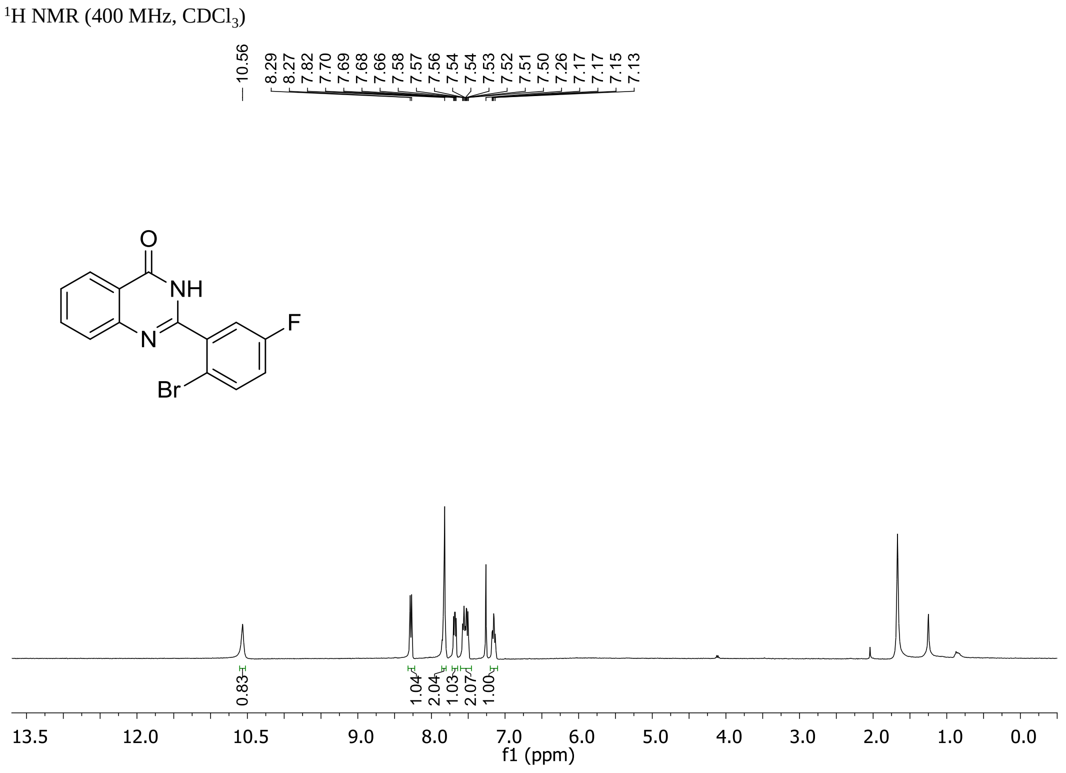

Figure S38. ^{1}H NMR spectra of 2-(2-bromo-5-fluorophenyl)quinazolin-4(3*H*)-one (**3s**).

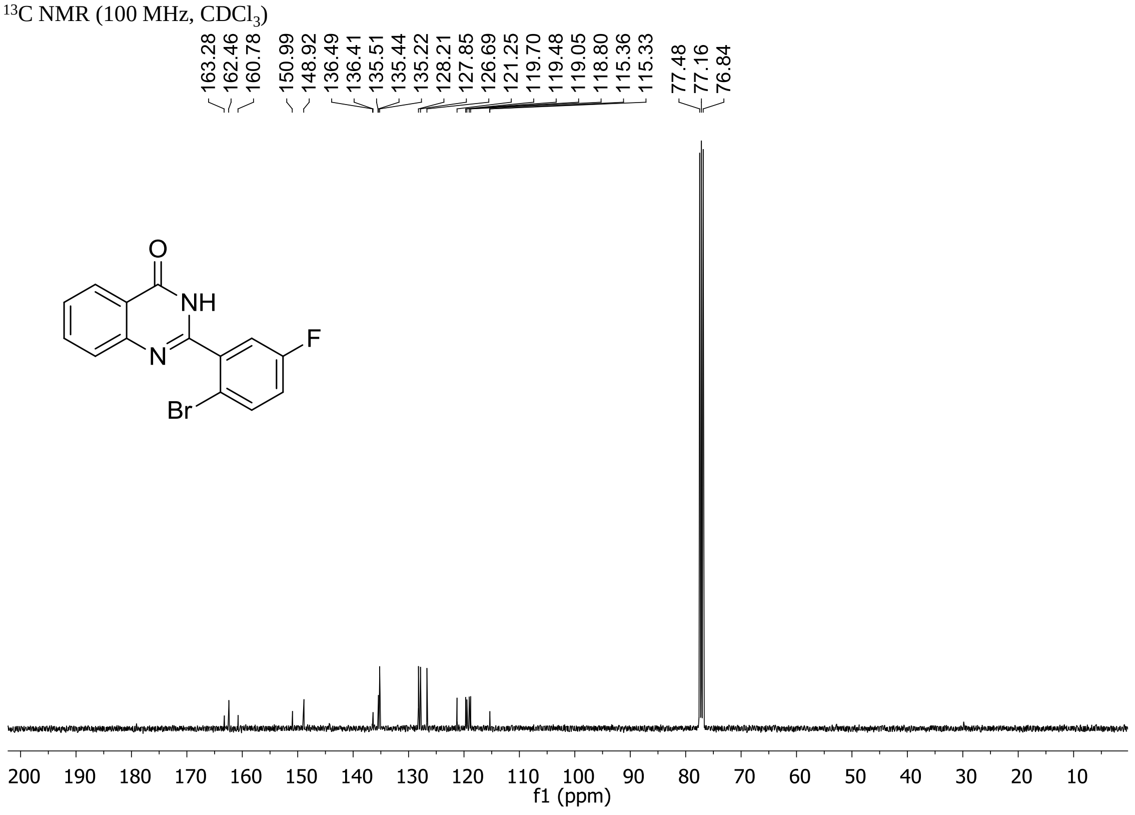

Figure S39. ^{13}C NMR spectra of 2-(2-bromo-5-fluorophenyl)quinazolin-4(3*H*)-one (**3s**).

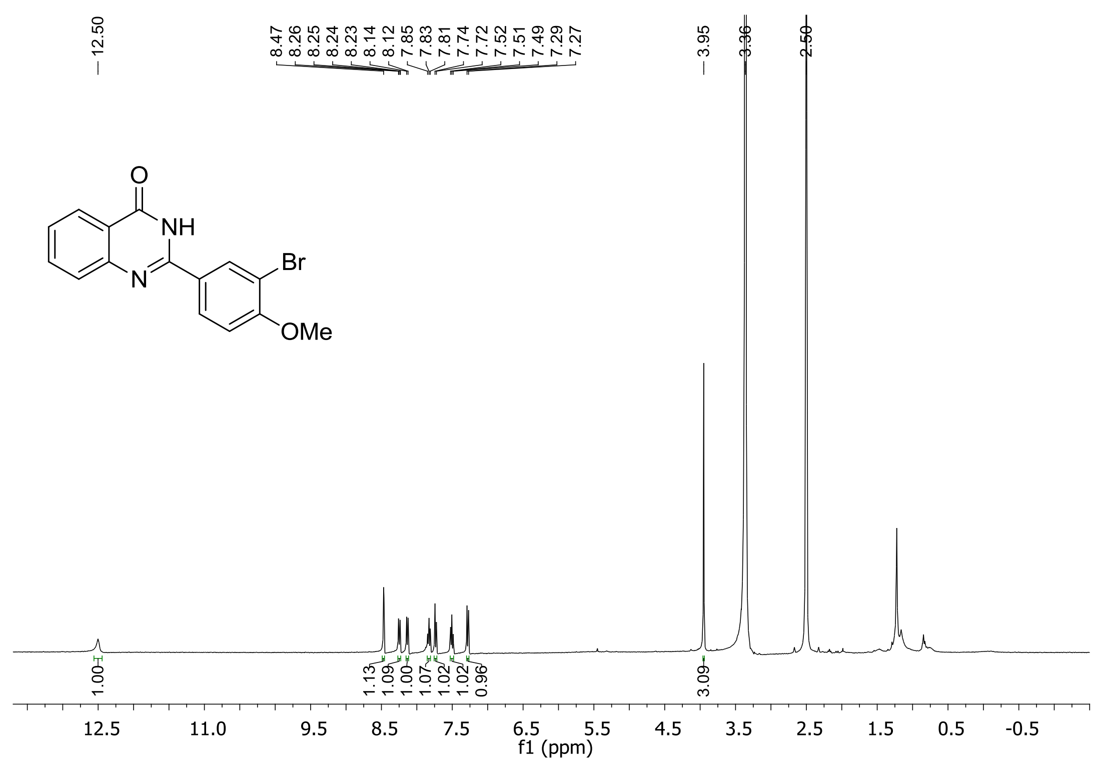

Figure S40. ^{1}H NMR spectra of 2-(3-bromo-4-methoxyphenyl)quinazolin-4(3*H*)-one (**3t**).

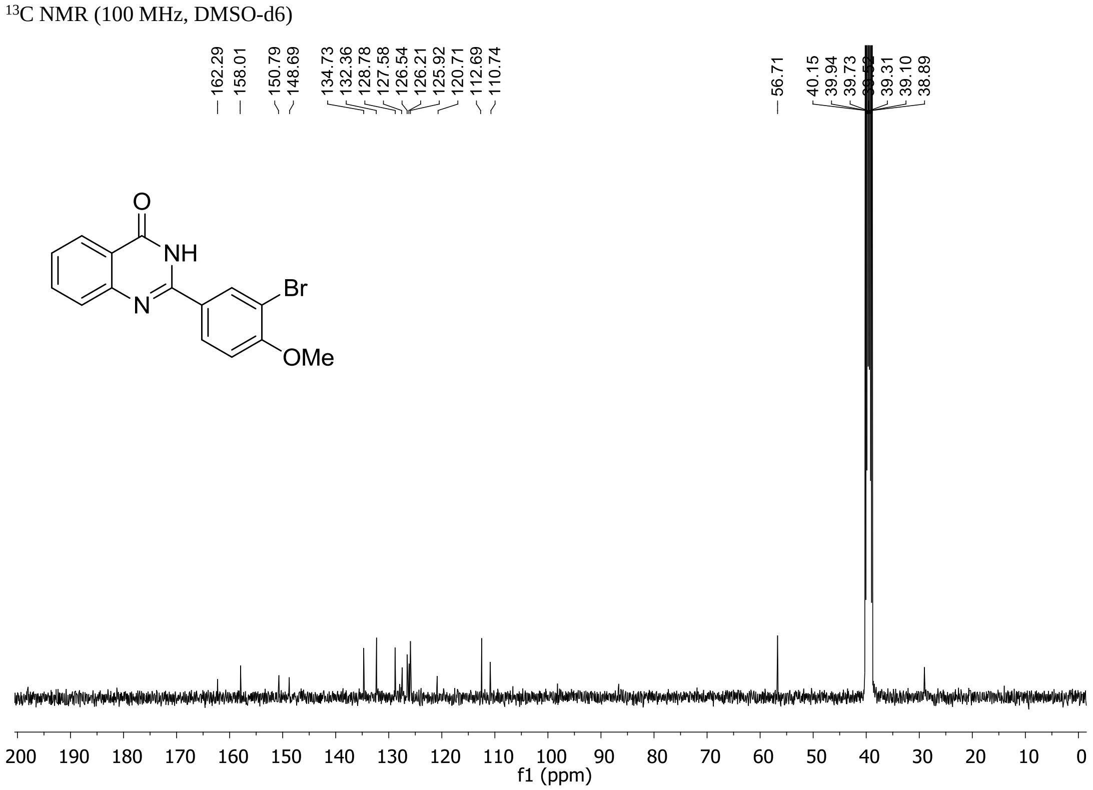

Figure S41. ^{13}C NMR spectra of 2-(3-bromo-4-methoxyphenyl)quinazolin-4(3*H*)-one (**3t**).

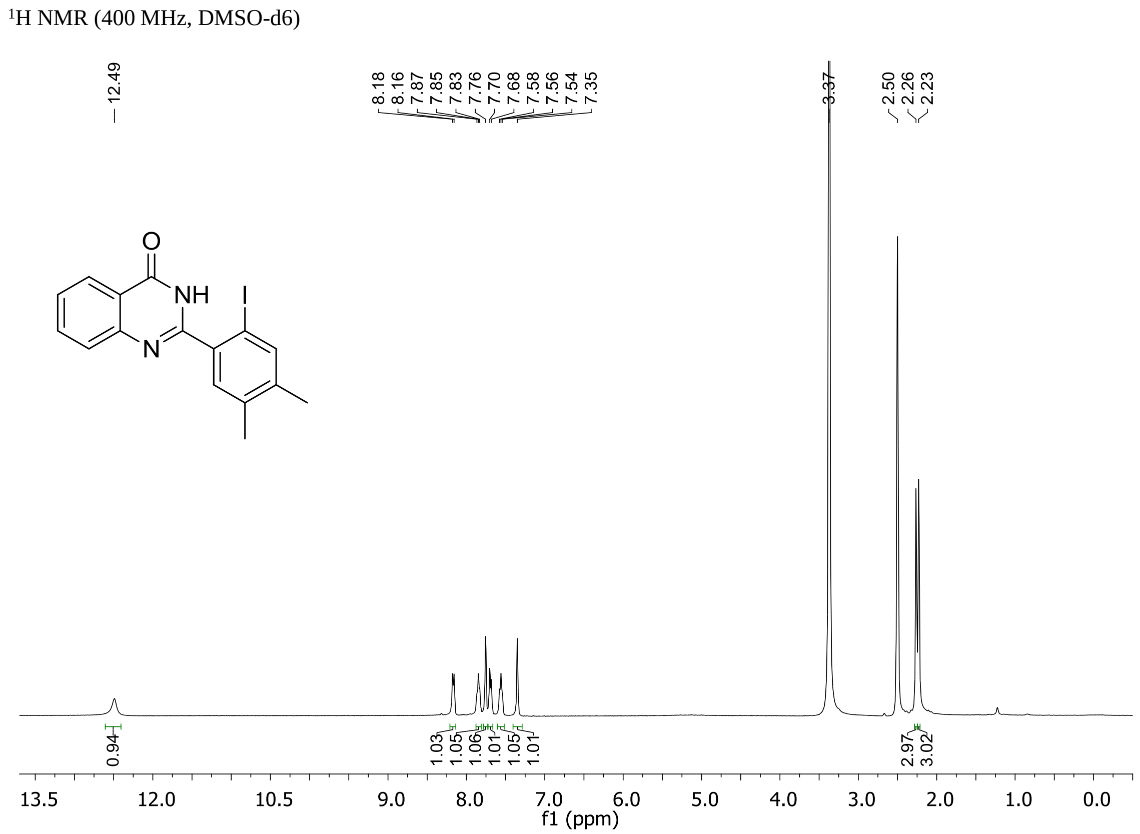

Figure S42. ^{1}H NMR spectra of 2-(2-iodo-4,5-dimethylphenyl)quinazolin-4(3*H*)-one (**3u**).

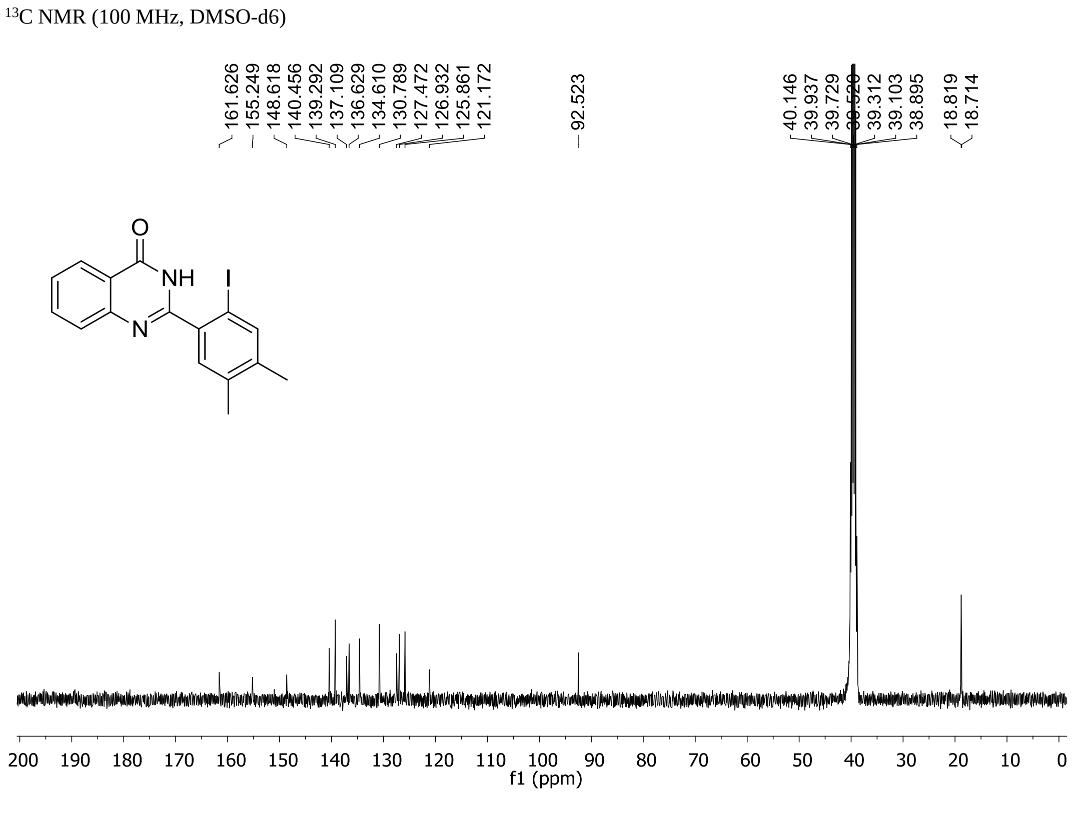

Figure S43. ^{13}C NMR spectra of 2-(2-iodo-4,5-dimethylphenyl)quinazolin-4(3*H*)-one (**3u**).

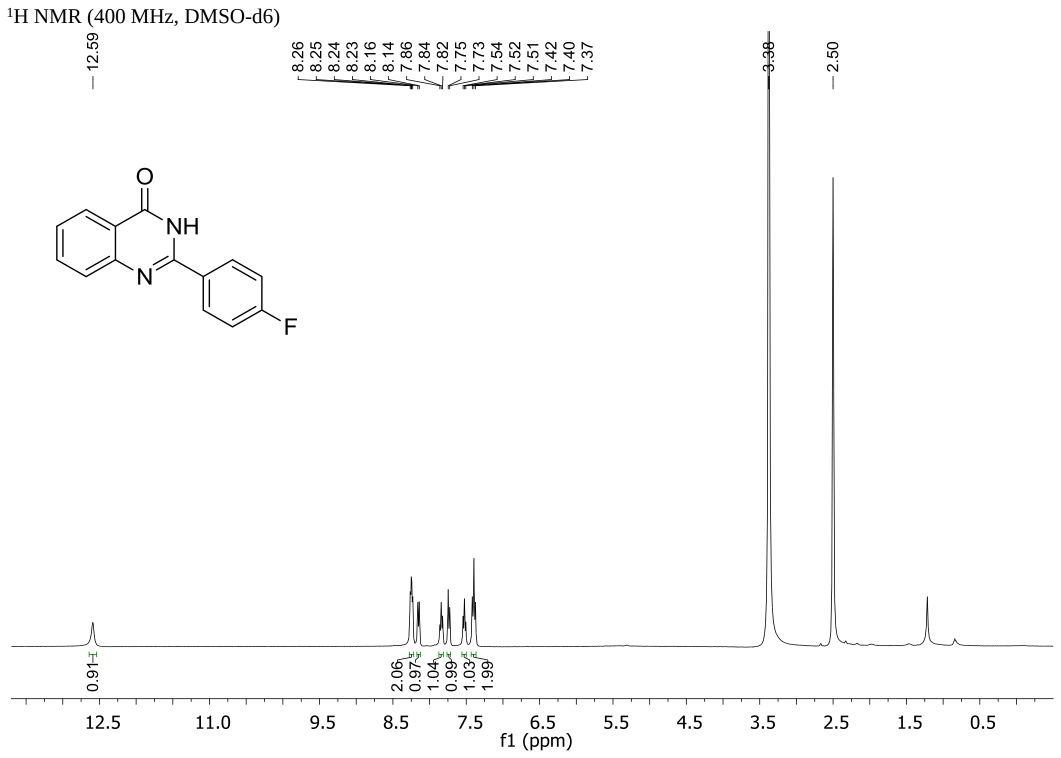

Figure S44. ^{1}H NMR spectra of 2-(4-fluorophenyl)quinazolin-4(3*H*)-one (**3v**).

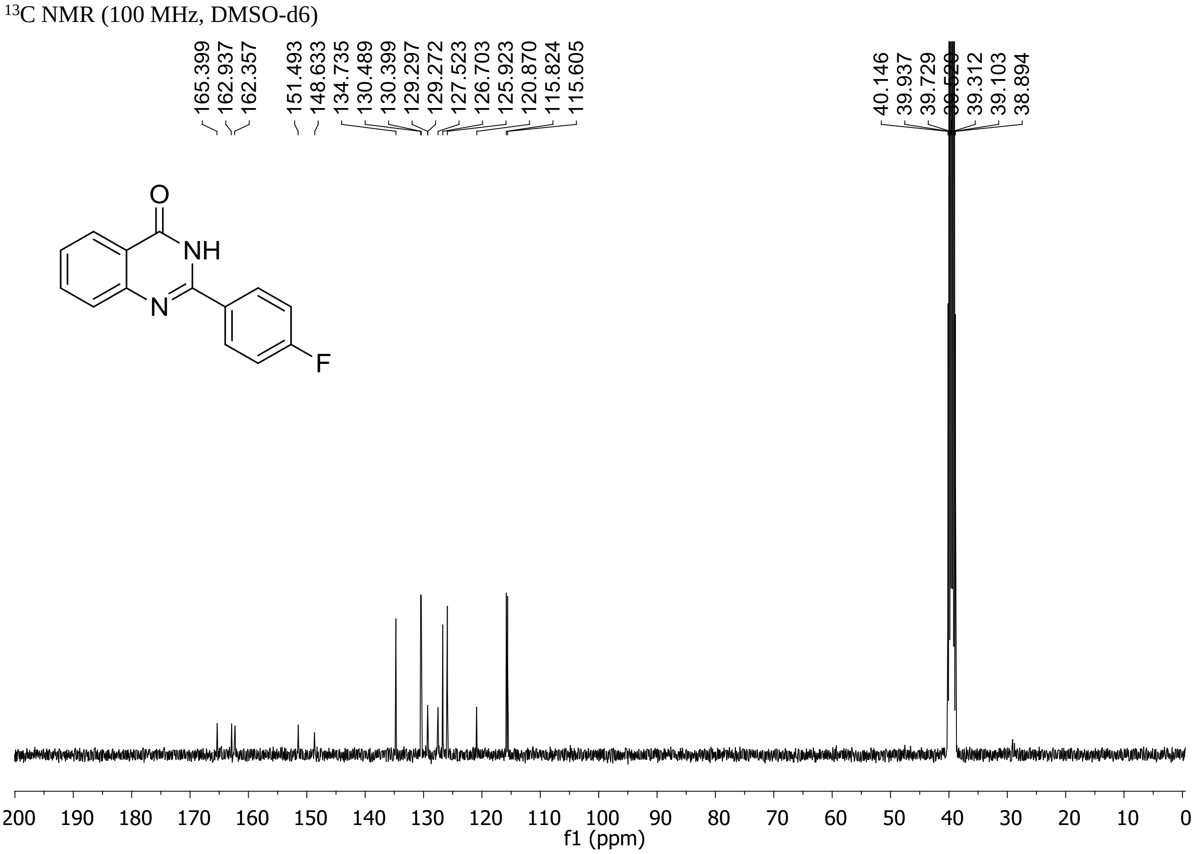

Figure S45. ^{13}C NMR spectra of 2-(4-fluorophenyl)quinazolin-4(3*H*)-one (**3v**).

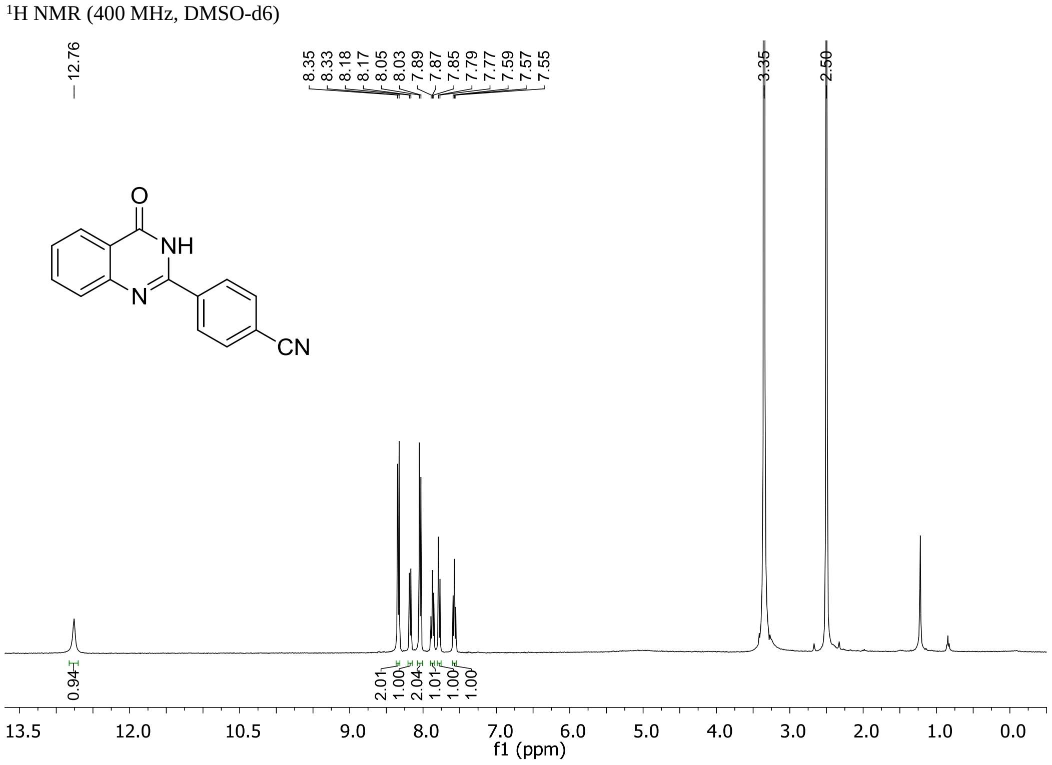

Figure S46. ^{1}H NMR spectra of 4-(4-oxo-3,4-dihydroquinazolin-2-yl)benzonitrile (**3w**).

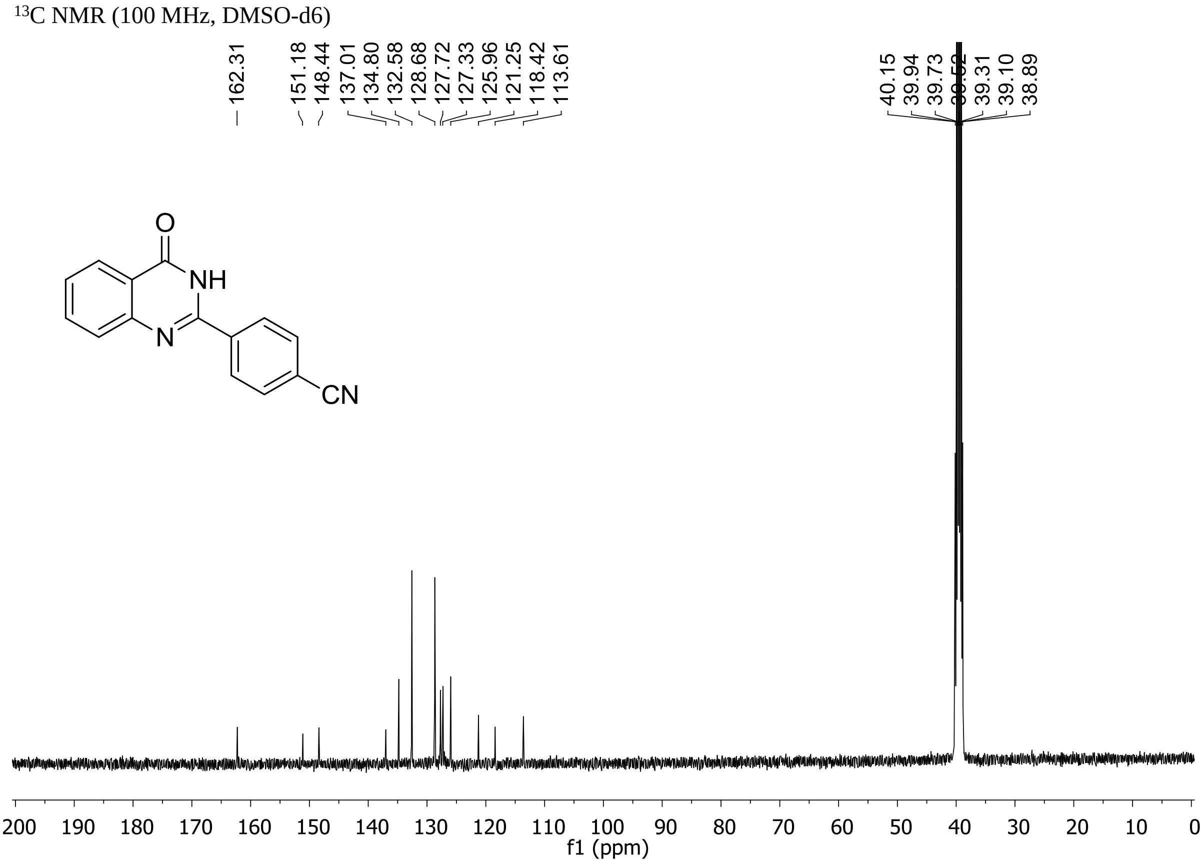

Figure S47. ^{13}C NMR spectra of 4-(4-oxo-3,4-dihydroquinazolin-2-yl)benzonitrile (**3w**).

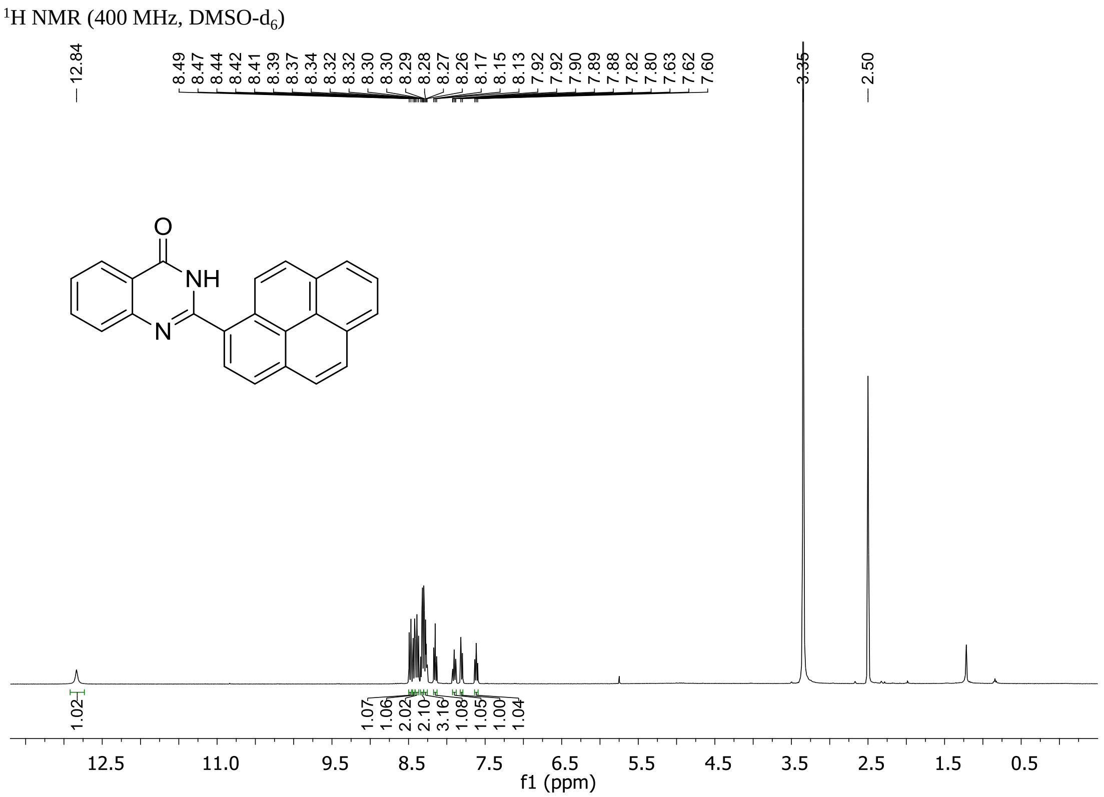

Figure S48. ^{1}H NMR spectra of 2-(pyren-1-yl)quinazolin-4(3*H*)-one (**3x**).

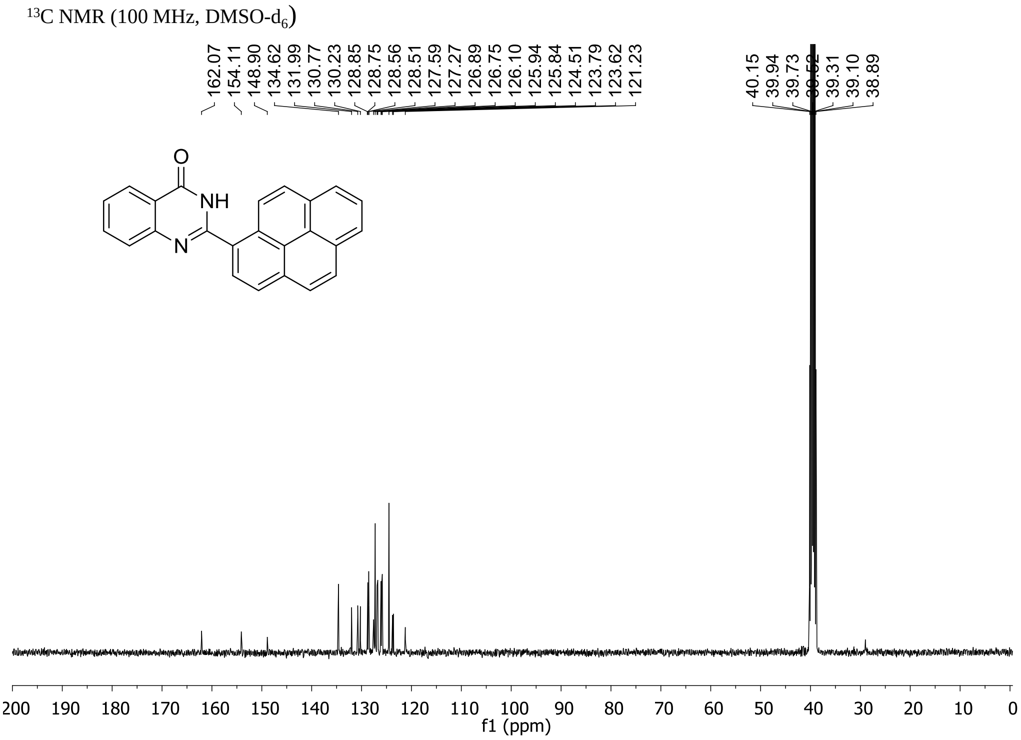

Figure S49. ^{13}C NMR spectra of 2-(pyren-1-yl)quinazolin-4(3*H*)-one (**3x**).

^{1}H NMR (400 MHz, $CDCl_3$)

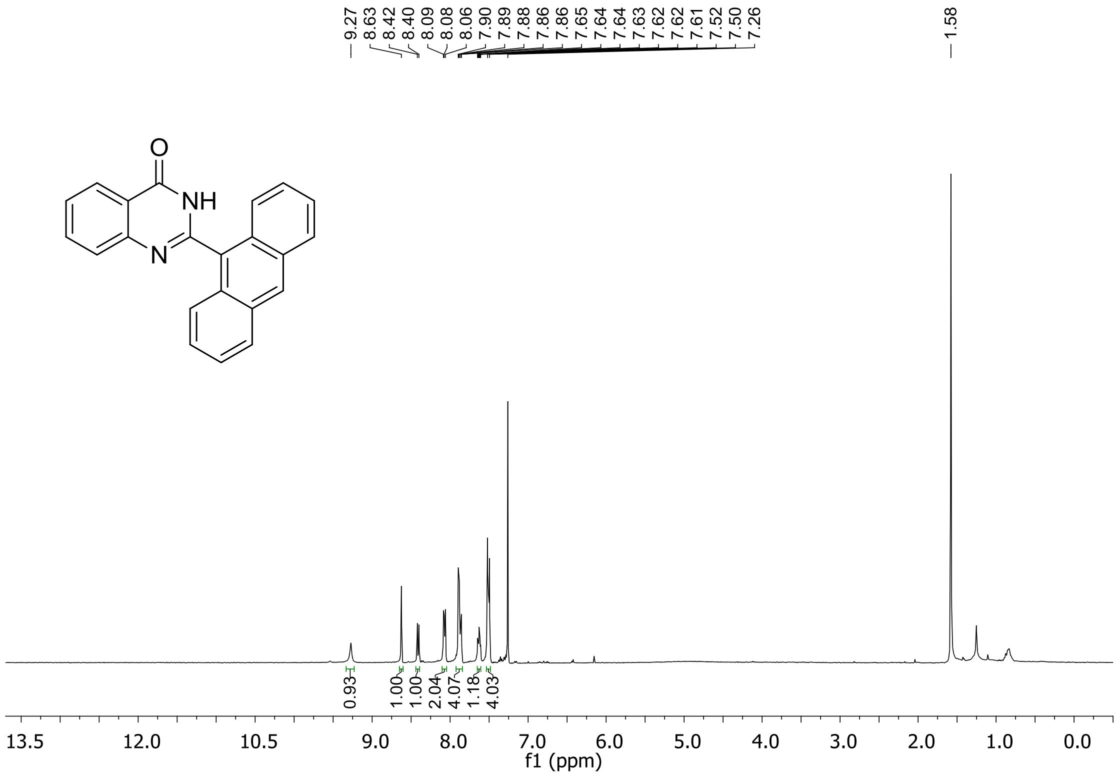

Figure S50. ^{1}H NMR spectra of 2-(anthracen-9-yl)quinazolin-4(3*H*)-one (**3y**).

^{13}C NMR (100 MHz, $CDCl_3$)

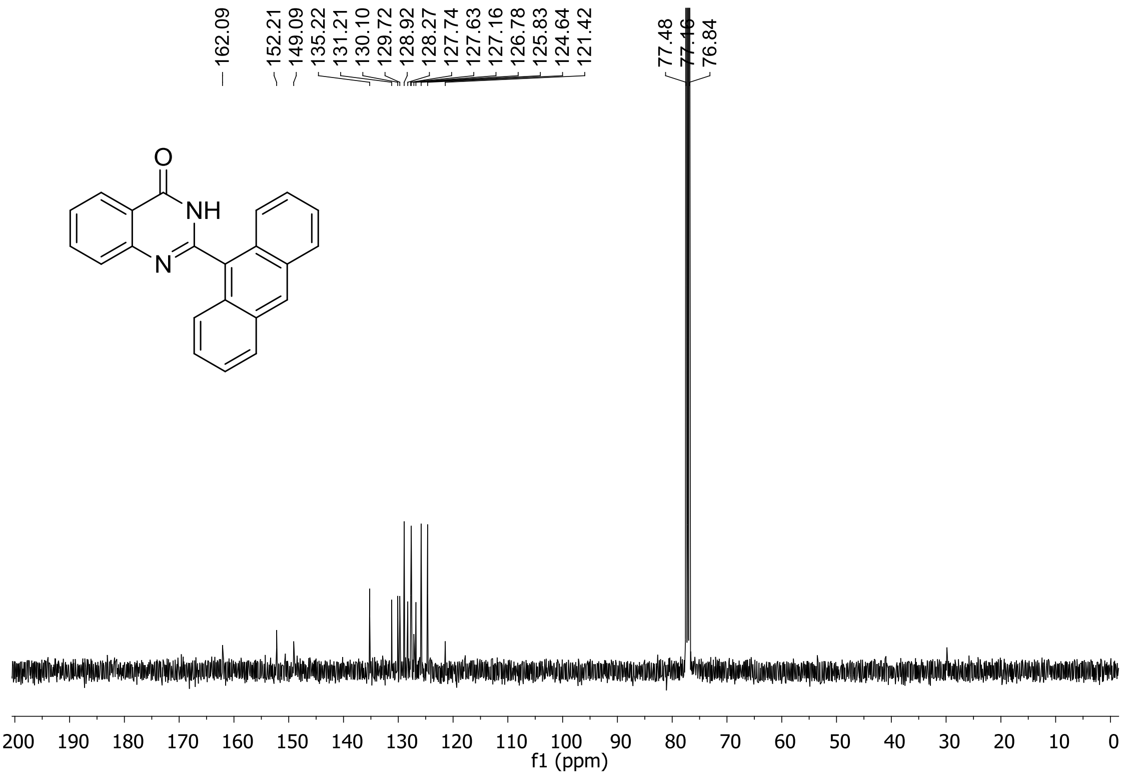

Figure S51. ^{13}C NMR spectra of 2-(anthracen-9-yl)quinazolin-4(3*H*)-one (**3y**).
